# Kidneys on the Frontline: Nephrologists Tackling the Wilds of Acute Kidney Injury in Trauma Patients—From Pathophysiology to Early Biomarkers

**DOI:** 10.3390/diagnostics15192438

**Published:** 2025-09-25

**Authors:** Merita Rroji, Marsida Kasa, Nereida Spahia, Saimir Kuci, Alfred Ibrahimi, Hektor Sula

**Affiliations:** 1 Department of Nephrology, University of Medicine, 1005 Tirana, Albania; edaspahia01@gmail.com; 2 Department of Internal Medicine, University Hospital of Trauma, 1000 Tirana, Albania; marsi.med@hotmail.com; 3 Department of Intensive Care, University of Medicine, 1005 Tirana, Albania; saimirkuci@gmail.com (S.K.); alfredibrahimi@hotmail.com (A.I.); hektorsula@yahoo.com (H.S.)

**Keywords:** trauma-related AKI (TRAKI), functional renal indices, urinary sodium (UNa), fractional excretion of potassium (FEK)

## Abstract

Acute kidney injury (AKI) is a frequent and severe complication in trauma patients, affecting up to 28% of intensive care unit (ICU) admissions and contributing significantly to morbidity, mortality, and long-term renal impairment. Trauma-related AKI (TRAKI) arises from diverse mechanisms, including hemorrhagic shock, ischemia–reperfusion injury, systemic inflammation, rhabdomyolysis, nephrotoxicity, and complex organ crosstalk involving the brain, lungs, and abdomen. Pathophysiologically, TRAKI involves early disruption of the glomerular filtration barrier, tubular epithelial injury, and renal microvascular dysfunction. Inflammatory cascades, oxidative stress, immune thrombosis, and maladaptive repair mechanisms mediate these injuries. Trauma-related rhabdomyolysis and exposure to contrast agents or nephrotoxic drugs further exacerbate renal stress, particularly in patients with pre-existing comorbidities. Traditional markers such as serum creatinine (sCr) are late indicators of kidney damage and lack specificity. Emerging structural and stress response biomarkers—such as neutrophil gelatinase-associated lipocalin (NGAL), kidney injury molecule 1 (KIM-1), liver-type fatty acid-binding protein (L-FABP), interleukin-18 (IL-18), C-C motif chemokine ligand 14 (CCL14), Dickkopf-3 (DKK3), and the U.S. Food and Drug Administration (FDA)-approved tissue inhibitor of metalloproteinases-2 × insulin-like growth factor-binding protein 7 (TIMP-2 × IGFBP-7)—allow earlier detection of subclinical AKI and better predict progression and the need for renal replacement therapy. Together, functional indices like urinary sodium and fractional potassium excretion reflect early microcirculatory stress and add clinical value. In parallel, risk stratification tools, including the Renal Angina Index (RAI), the McMahon score, and the Haines model, enable the early identification of high-risk patients and help tailor nephroprotective strategies. Together, these biomarkers and risk models shift from passive AKI recognition to proactive, personalized management. A new paradigm that integrates biomarker-guided diagnostics and dynamic clinical scoring into trauma care promises to reduce AKI burden and improve renal outcomes in this critically ill population.

## 1. Overview of the Incidence Rates and Significance of Acute Kidney Injury (AKI) in Trauma Patients

Traumatic injury, accounting for an estimated 8–10% of all global fatalities [1], is recognized as a considerable public health concern and remains a leading cause of death worldwide [2,3]. Although direct kidney trauma is relatively uncommon, AKI secondary to extrarenal injuries occurs frequently, with reported incidences ranging from 0.8% [1] to about 67%, depending on the diagnostic criteria used [4]. These variations largely stem from differences in definitions—KDIGO (Kidney Disease: Improving Global Outcomes) versus RIFLE (Risk, Injury, Failure, Loss of kidney function, and End-stage kidney disease). KDIGO’s broader thresholds detect more subtle declines in renal function, thereby yielding higher AKI rates, whereas RIFLE’s stricter criteria result in lower classifications.

Beyond trauma contexts, up to 50% of critically ill patients develop AKI, significantly heightening both short- and long-term morbidity and mortality [5,6,7,8,9,10,11]. Mortality varies from 7% in mild cases of AKI to as high as 69% in its most severe stages [9,10,12]. In populations specifically affected by trauma, AKI remains a prominent clinical concern. Among individuals who survive the initial injury, 20–28% admitted to intensive care units (ICUs) develop AKI [13,14,15,16]. For patients with traumatic brain injury, AKI incidence ranges from 12% to 39.3%, peaking within the first two days of admission [17,18,19,20].

In addition, a systematic review of data from more than 25,000 trauma ICU patients (2004–2018) reported a 24% incidence of AKI, with onset typically within three days (range: 1–6) [14]. Over half of these cases were categorized as mild, whereas approximately 10% required renal replacement therapy (RRT)—equating to 2% of the total patient population [14,15,16]. Notably, 96% of those affected recovered renal function, reflecting the robust renal reserve often observed in trauma populations without pre-existing chronic conditions [14].

It is necessary to underline that heightened susceptibility to AKI extends beyond direct renal trauma and encompasses a range of contributing factors, including hypotension, hemorrhagic shock, massive transfusions, rhabdomyolysis, abdominal compartment syndrome, elevated injury severity scores, infectious complications, organ crosstalk, and inflammation. Additionally, diminished renal functional reserve—frequently observed in older adults and individuals with pre-existing chronic kidney disease (CKD), diabetes, and hypertension—further increases the risk. Exposure to nephrotoxic agents and medications exacerbates this vulnerability [12,15,21]. Moreover, contrast-induced acute kidney injury (CI-AKI) is a prominent concern within the trauma setting, as reported in multiple investigations [22,23,24,25]. Against this backdrop, the present review was designed as a narrative synthesis of the current knowledge on trauma-related acute kidney injury (TRAKI). It aims to provide a panoramic overview that merges epidemiological data, mechanistic pathways, and emerging biomarkers, thereby offering clinicians and researchers a comprehensive perspective on how fundamental pathophysiological insights may inform diagnosis, risk stratification, and prevention. To guide this effort, we organized the evidence thematically around incidence, pathophysiology, organ crosstalk, emerging biomarkers, and predictive models in critically ill trauma patients. We performed a targeted literature search in PubMed/MEDLINE, Embase, and the Cochrane Library, encompassing studies published between January 2000 and January 2025. The search terms integrated acute kidney injury, trauma, intensive care, pathophysiology, organ crosstalk, and diagnostic/prognostic biomarkers. We included narrative reviews, systematic reviews, guidelines, and both observational and interventional studies, giving priority to research on adult, trauma populations with mechanistic or clinical relevance. Reference lists of key articles were also reviewed to identify additional evidence.

## 2. Breaking Down Trauma-Related AKI

### 2.1. Trauma-Induced Shock and Its Impact on Renal Function: Mechanisms of Injury and Recovery

#### 2.1.1. Hemodynamic Instability and Shock

Trauma can precipitate various types of shock, most deriving from an imbalance between oxygen delivery and utilization. hypovolemic, cardiogenic, or obstructive shock, such as that seen in myocardial infarction or pulmonary embolism, reduces cardiac output and promotes systemic vasoconstriction, thereby curtailing oxygen transport [26]. In contrast, distributive shock, typified by septic shock, occurs from a decline in vascular resistance accompanied by aberrant tissue perfusion [27]. The physiological reaction to trauma and hemorrhage generally comprises an initial hypometabolic phase, followed by a hypermetabolic phase once tissue perfusion is restored, which can trigger ischemia–reperfusion injury (IRI) through the release of reactive oxygen species (ROS) and reactive nitrogen species (RNS) [28,29]. Prolonged inadequate perfusion can lead to ischemic tubular necrosis and AKI, even in the absence of overt structural kidney damage. Furthermore, renal blood flow may remain insufficient following resuscitation, perpetuating hypoperfusion and diminishing the glomerular filtration rate [30,31].

Recent findings introduce the concept of renal microcirculatory stress (RMS) to describe scenarios in which sodium-retaining mechanisms are activated despite normal or increased renal artery blood flow, as observed in hyperdynamic states such as sepsis [32]. These hemodynamic disorders lower glomerular filtration pressure and promote sodium retention, decreasing urinary sodium (UNa) levels. Making it possible to incorporate a broader perspective than the traditional “pre-renal paradigm,” RMS highlights glomerular hemodynamic conditions that can contribute to AKI even without structural kidney injury. Renal microcirculatory dysfunction—a critical precursor to AKI—is highly relevant in the context of trauma-induced AKI. Hypoxemia and tissue injury, as seen in IRI and shock, activate leukocytes and promote the release of inflammatory mediators, which drive systemic inflammation and disrupt renal microvascular homeostasis [33].

Apart from transient renal hypoperfusion alone, additional contributors, including immune activation and metabolic shifts following trauma, further heighten susceptibility. Traumatic hypoxia, ischemic insults, and compromised perfusion each play a critical role in inciting renal injury, inflammation, and disruption of the blood–urine barrier [34]. Beyond the direct effect of trauma, anesthesia and surgical interventions can worsen renal dysfunction through myocardial depression, vasodilation, and fluid losses [35].

Meanwhile, elevated central venous pressures, fluid overload, and increased intra-abdominal pressure reduce renal perfusion pressure and hinder venous outflow, promoting interstitial edema and exacerbating hypoxia. These conditions amplify metabolic stress by increasing oxygen demand while limiting delivery, which elevates the likelihood of AKI [31,36]. Ultimately, AKI emerges from a cascade of cellular derangements merging on the renal microcirculation. Adequate oxygen delivery and utilization at both microcirculatory and mitochondrial levels are crucial for renal viability. However, the kidney’s complex microvascular architecture, notable energy needs, and borderline hypoxemic baseline render it exceptionally prone to injury [37]. In trauma and other pathological states, disruptions in renal microcirculation lead to imbalances among nitric oxide, ROS, and oxygen regulation, resulting in hypoxemia and oxidative stress that aggravate kidney damage [38,39].

#### 2.1.2. Immune Thrombosis and Inflammatory Pathways in Trauma

Drivers of renal injury: The systemic inflammatory response to trauma and hemorrhagic shock significantly contributes to kidney injury. Following a traumatic event, the immune system activates within minutes through the autonomic nervous system and fluid-phase processes [40,41]. At both internal and external injury sites, a rapid phenomenon termed immune thrombosis takes place. This ancient survival mechanism not only helps prevent blood loss but also limits the spread of pathogens [41]. During immune thrombosis, injured tissues release neo-antigens and damage-associated molecular patterns (DAMPs)—including nucleosomes, histones, RNA, ATP, mitochondrial DNA, and uric acid—which activate coagulation and complement pathways, rapidly consuming clotting factors [41,42]. Elevated levels of DAMPs, such as high-mobility group protein B1 (HMGB1), often occur in traumatic hemorrhagic shock, while pathogen-associated molecular patterns (PAMPs), like endotoxins, rise in septic shock [43,44].

Cells in both tissues and circulation detect these danger signals, prompting pro- and anti-inflammatory responses through a broad spectrum of cytokines and chemokines. In trauma patients, those who develop AKI show significantly increased early concentrations of both anti-inflammatory markers—such as interleukin-1 receptor antagonist (IL-1Ra)—and pro-inflammatory mediators, including IL-6, IL-8, and MCP1 (CCL2) [45]. Moreover, individuals with AKI and subsequent infections exhibit higher levels of additional chemokines, such as CXCL1 and CCL4 [46]. These chemokines recruit leukocytes, which then release cytokines, perform phagocytosis, generate oxidative bursts, and produce proteases. Leukocytes also form neutrophil and macrophage extracellular traps that eliminate damaged tissue and pathogens while simultaneously facilitating tissue repair [41].

The coagulation system, which directs hemostasis, depends on over a trillion platelets to repair injured vessels and restore oxygenation to damaged tissues. In the aftermath of trauma, platelets rapidly adhere to injury sites, activating the coagulation cascade. Thrombin and fibrin are produced, forming clots that stabilize wounds and limit further hemorrhage [47].

However, in the setting of severe trauma or hemorrhagic shock, this finely tuned system can become overactive or exhaust its resources, leading to life-threatening trauma-induced coagulopathy. Even tissues distant from the initial injury may be impacted, as inflammation alters the endothelium of blood vessels—thereby promoting clot formation, oxidative stress, and further inflammatory responses [48].

### 2.2. Integrative Pathophysiological Mechanisms of Trauma-Induced Kidney Injury: Dysregulation of Glomerular, Tubular, and Endothelial Components

Post-trauma kidney immune reactions are not well understood, with most insights coming from studies on IRI and hemorrhagic shock. These conditions lead to damage across multiple organs, including the kidneys. The immune response following trauma triggers pro-inflammatory changes in the kidneys, particularly in the glomeruli, resulting in increased inflammatory mediators that pass through the glomerular filter.

Glomerular damage: The glomerular filter is a unique structure in the kidney that depends on harmonized interactions among mesangial cells, glomerular endothelial cells (GECs), and podocytes to ensure selective permeability. Water and small- to mid-sized molecules pass freely through this filtration barrier, whereas larger proteins, such as albumin, are typically restricted. Each cell type contributes different functions to the filtration process, and any disruption in their interplay can precipitate glomerular injury, proteinuria, and AKI [49].

Mesangial cells, located centrally within the glomerulus, provide structural support and regulate capillary loop tone, extracellular matrix production, and the release of inflammatory mediators. These cells also generate nitric oxide (NO) and ROS and engage in phagocytosis of immune complexes and debris to maintain glomerular homeostasis [50]. However, under pathological conditions such as trauma, burns, or sepsis, mesangial cells respond to damage-associated molecular patterns and pathogen-associated molecular patterns by producing pro-inflammatory cytokines, including interleukin-1β (IL-1β), tumor necrosis factor (TNF), and monocyte chemoattractant protein-1 (MCP-1) [51,52,53]. This response amplifies local and systemic inflammation, alters vascular resistance, and contributes to renal dysfunction.

GECs form the innermost filtration layer and are characterized by their fenestrations, which enable the efficient formation of ultrafiltrate. These cells regulate vascular tone, permeability, and the trafficking of immune cells, thereby preserving homeostasis under various environmental stresses. However, GECs may adopt a pro-inflammatory phenotype in the context of trauma or sepsis, which can impair endothelial function and increase vascular permeability, thereby compromising the filtration barrier and promoting proteinuria. The additional loss of the endothelial glycocalyx further disrupts barrier integrity and exacerbates glomerular injury [54,55].

Podocytes, specialized epithelial cells that encase the glomerular capillaries, are crucial for maintaining the filtration barrier’s selectivity and structural integrity [56,57]. Their foot processes and slit diaphragms attach to the glomerular basement membrane (GBM), dynamically adapting to fluctuations in filtration pressure through changes in the actin cytoskeleton. However, when faced with inflammatory mediators, trauma, or sepsis, podocytes become damaged, leading to foot process effacement, slit diaphragm disruption, and proteinuria. Given their limited regenerative capacity, podocyte injury is particularly deleterious, potentially initiating a cycle of glomerular injury that can progress to CKD [58].

The functional interplay among mesangial cells, GECs, and podocytes is important for glomerular filtration yet is disrupted in conditions such as trauma, burns, and sepsis, affecting cellular stress, inflammation, and oxidative damage. Mesangial cells drive local inflammation and profound regulatory changes; endothelial cells lose their capacity to maintain vascular homeostasis, and podocyte integrity deteriorates, culminating in AKI. Inflammatory mediators—such as IL-1β, TNF, and NO—alongside complement activation and glycocalyx shedding collectively intensify the injury [58,59].

A thorough understanding of these cellular mechanisms and their responses to injury is essential for developing effective therapeutic strategies to mitigate inflammation, oxidative stress, and tissue damage, thereby maintaining the glomerular barrier and preventing renal dysfunction.

Tubular epithelial cell (TEC) vulnerability: TECs play a pivotal role in renal function; however, they are particularly vulnerable to injury when inflammatory processes extend from the glomerulus into the tubule–interstitium. Within the tubular compartment, reduced blood flow and oxygen deprivation are primary drivers of AKI, giving rise to cellular changes such as swelling, loss of polarity, and necrosis [60]. TECs exhibit high metabolic activity in an environment characterized by low baseline oxygen, rendering them susceptible to damage, particularly under traumatic conditions [61]. Upon stress or injury, TECs not only produce cytokines, chemokines, and DAMPs but also serve as targets for these inflammatory mediators [51,62].

In an important trauma, acute kidney injury arises from a combined ischemic and toxic insult with a segmental pattern of vulnerability. The proximal tubule, particularly the S3 segment in the outer medulla, sustains the earliest and most severe injury from hemorrhagic shock-related ischemia–reperfusion and nephrotoxins typical of trauma care (e.g., myoglobin from rhabdomyolysis, iodinated contrast), leading to loss of polarity, brush-border disruption, and necrosis. The medullary thick ascending limb is also highly susceptible: its high metabolic demand within a hypoxic region makes it prone to hypoperfusion from shock, vasoconstrictors, or abdominal compartment syndrome and to oxidative stress during reperfusion, which impairs its concentrating ability and structure [60]. In contrast, the distal convoluted tubule and collecting duct show relative resistance to direct ischemia but contribute critically to downstream pathology, as Tamm–Horsfall protein, myoglobin, and necrotic debris aggregate into obstructive casts that aggravate upstream tubular stress and precipitate electrolyte and acid–base disturbances [63]. Thus, trauma-related AKI typically manifests as a mixed lesion: proximal necrosis predominates, medullary hypoxic stress amplifies dysfunction, and distal obstruction compounds filtration failure.

Moreover, toll-like receptors (TLR2 and TLR4) on the luminal surface of TECs become upregulated following IRI, improving the recognition of DAMPs and PAMPs. This amplification of the inflammatory response is further evidenced by the contribution of TECs to cytokine production, as demonstrated by transcriptomic studies in murine models of IRI, which have identified the activation of IL-10, IL-6, TLR, and JAK-STAT signaling pathways [51,64,65]. Moreover, IL-34 secreted by TECs can initiate macrophage-mediated tissue damage, while hypoxia-inducible factors (HIFs), particularly HIF1α, accumulate under hypoxic conditions and promote additional macrophage recruitment. In AKI, reduced clearance of cytokines such as HMGB1, IL-6, IL-10, and TNF elevates local and systemic inflammatory activity [66].

Although circulating mitochondrial DNA (mtDNA) acts as a DAMP and an inflammatory trigger in trauma, its specific role in post-trauma AKI remains limited. However, urinary mtDNA levels correlate with an increased albumin-to-creatinine ratio and elicit pro-inflammatory responses in TECs [67]. Another key marker, kidney injury molecule 1 (KIM-1), is highly upregulated in TECs in response to ischemic or toxic injury during AKI and is shed into the proximal tubule. The degree of KIM-1 expression correlates with injury severity, notably in combat-related cases. By detecting apoptosis-related epitopes and adopting phagocytic functions, KIM-1-expressing TECs facilitate the clearance of necrotic and apoptotic cells [68].

In addition to KIM-1, Dickkopf-3 (DKK3) is secreted in urine following renal stress and has been associated with the prediction of postoperative AKI [69]. DKK3 is upregulated during tubulointerstitial stress, chronic injury, and fibrogenic signaling, acting as a profibrotic mediator contributing to CKD progression [70]. Maladaptive repair after AKI involves tubular cell senescence, G2/M cell cycle arrest, and persistent inflammation, which promotes fibrosis and accelerates kidney aging [71]. Biomarkers such as tissue inhibitor of metalloproteinases-2 (TIMP-2) and insulin-like growth factor-binding protein 7 (IGFBP7) are also produced by tubular epithelial cells in response to early cellular stress [72,73]. Despite a lack of distinct progenitor cells, residual TECs can repopulate the renal epithelium after trauma, underscoring their regenerative potential [74,75].

Neutrophils and macrophages further modulate kidney injury in the post-trauma setting. Neutrophils migrate rapidly to the kidney and secrete pro-inflammatory signals, yet insufficient recruitment can paradoxically contribute to damage in distant organs. Macrophages, meanwhile, clear injured tissue and facilitate recovery but also participate in early inflammatory processes [76].

Vascular and endothelial dysfunction: Trauma also affects the renal vasculature, with alterations in blood flow and endothelial function contributing to additional injury. Pericytes, which are critical for maintaining vascular and endothelial homeostasis, secrete angiogenic factors such as angiopoietin, platelet-derived growth factor (PDGF), sphingosine-1-phosphate (S1P), and vascular endothelial growth factor (VEGF). In AKI, pericytes become activated, detach from the vasculature, and exhibit myofibroblast-like properties, particularly in the context of IRI or ureteral obstruction. Trauma and shock induce the release of DAMPs, proteases, and other inflammatory mediators, leading to the degradation of the endothelial glycocalyx—a key feature of trauma-induced endotheliopathy—and resulting in the dysfunction of the renal microcirculation [77,78]. Thrombin further compromises the endothelial barrier within the renal microvasculature [33].

During AKI, the reduced expression of endothelial nitric oxide synthase (eNOS) limits NO production, impairing vascular function and contributing to renal microcirculatory dysfunction. Moreover, a concurrent decrease in prostacyclin—like NO, a platelet aggregation inhibitor—further promotes a pro-coagulant milieu [33]. DAMPs, such as uric acid, stimulate the release of Weibel–Palade bodies, which secrete mediators including von Willebrand factor (vWF) and P-selectin, thereby promoting platelet adhesion and activation. The angiopoietin–Tie2 signaling pathway, in which Tie2 refers to the endothelial tyrosine kinase receptor containing immunoglobulin-like and epidermal growth factor (EGF)-like domains, is a key regulator of vascular inflammation and permeability in the early stages of AKI. Elevated platelet-derived HMGB1 in trauma is associated with thrombus formation, reducing renal blood flow through increased leukocyte recruitment and red blood cell aggregation [79].

The angiopoietin–Tie2 (tyrosine kinase with immunoglobulin-like and Epidermal Growth Factor (EGF)-like domains 2) signaling pathway modulates vascular inflammation and permeability in early AKI. Angiopoietin-1 activates Tie2, exerting anti-inflammatory and anti-permeability effects, whereas angiopoietin-2 antagonizes Tie2, enhancing inflammation and endothelial leakage. Hemorrhagic shock raises concentrations of angiopoietin-2 and soluble Tie2, disrupting microcirculatory perfusion and diminishing Tie2 activity in the kidney; however, the Tie2 agonist vasculature provides renal protection in AKI [80,81].

The interplay between endothelial and epithelial cells in AKI can create a “vicious triad.” DNA from necrotic TECs induces platelet aggregation, which then interacts with neutrophils to form neutrophil extracellular traps (NETs). These NETs release toxic histones that further damage TECs. ROS upregulate TLR4 on endothelial cells within hours of IRI, followed by its increased expression on TECs [82]. HMGB1, either locally produced by TECs or derived systemically, signals through TLR4 in a time-dependent manner, initially prompting the expression of endothelial adhesion molecules and the recruitment of inflammatory cells, then eliciting cytokine production in TECs. Moreover, HMGB1-TLR4 binding on macrophages heightens IL-6 secretion, amplifying endotheliopathy and renal dysfunction [83].

To summarize, the glomerular filtration barrier, comprising mesangial, endothelial, and podocyte cells, together with tubular epithelial cells (TECs), collectively maintain renal function through tightly coordinated interactions. renal function through tightly coordinated interactions. In conditions such as trauma or sepsis, heightened inflammation and oxidative stress disrupt these structures, often leading to AKI. TECs are specifically vulnerable due to their high metabolic demands in a relatively hypoxic environment and react to injury by releasing cytokines and DAMPs, thus intensifying inflammation [51]. Concurrently, endothelial dysfunction, including glycocalyx degradation and impaired NO synthesis, fosters vascular inflammation, coagulopathy, and diminishing microperfusion [33].

**Figure 1:**
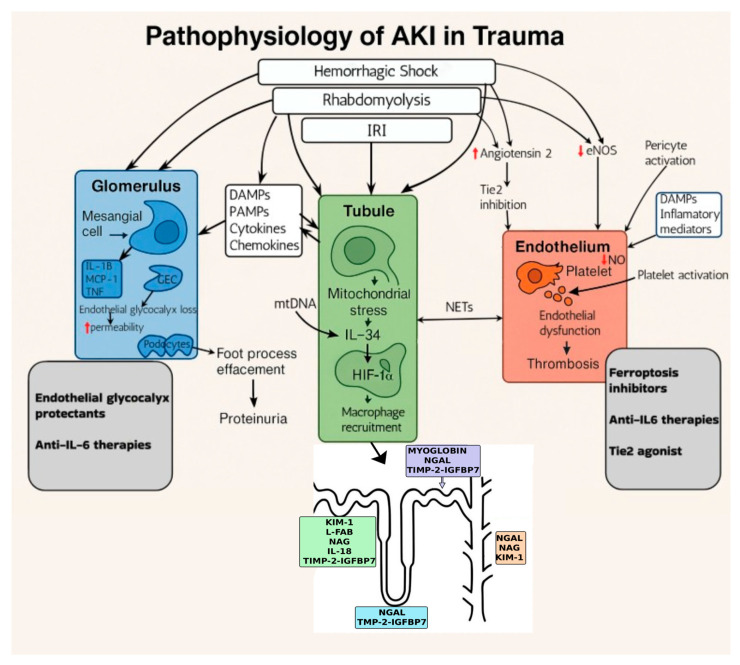
Pathophysiology of trauma-induced acute kidney injury: crosstalk among glomerular, tubular, and endothelial compartments. MCP-1, and TNF-α. Within the tubule (green), mitochondrial stress and release of mitochondrial DNA activate IL-34 and HIF-1α, recruiting macrophages; this involves the proximal tubule (S1, S2, S3) (KIM-1, L-FABP, NAG, IL-18, TIMP-2·IGFBP7), loop of Henle (NGAL, TIMP-2·IGFBP7), distal tubule (Myoglobin, NGAL, TIMP-2·IGFBP7), and collecting tubule (NGAL, NAG, KIM-1). Within the endothelium (red), reduced nitric oxide (NO) bioavailability, Tie2 inhibition, and platelet activation promote endothelial dysfunction and microvascular thrombosis. Inter-compartmental signals (arrows) include neutrophil extracellular traps (NETs) and angiotensin II-mediated pericyte activation, amplifying injury. The grey panels indicate emerging therapeutic strategies: endothelial glycocalyx protectants, anti-IL-6 agents, ferroptosis inhibitors, and Tie2 agonists. Abbreviations: eNOS, endothelial nitric oxide synthase; mtDNA, mitochondrial DNA.

The angiopoietin–Tie2 pathway further modulates endothelial permeability and inflammatory responses; its dysregulation underlies the progression of AKI [80,81]. Maladaptive TEC repair, characterized by cellular senescence, G2/M cell cycle arrest, and the release of profibrotic cytokines, exacerbates renal damage. Moreover, pericyte activation and platelet aggregation intersperse with these processes, generating a complex network of injury and inflammation that underscores the necessity for targeted therapeutic interventions to preserve renal integrity. A summary of the pathophysiology of AKI in trauma is presented in Figure 1.

## 3. Diverse Pathways to Renal Injury: Exploring AKI Triggers in the Trauma ICU

### 3.1. Trauma-Related Rhabdomyolysis (TRR)

Rhabdomyolysis is defined as the breakdown of skeletal muscle, marked by the release of cellular components, including myoglobin, sarcoplasmic enzymes, and electrolytes, into the extracellular fluid and bloodstream. Rhabdomyolysis can arise from either direct traumatic causes or metabolic origins, with the latter including factors such as exertion, muscle hypoxia, infections, metabolic and electrolyte disorders, drugs, toxins, and genetic defects.

TRR can be triggered by three main mechanisms: crush syndrome, hypoxia, and ischemia–reperfusion injury [84].

Crush syndrome (rhabdomyolysis from direct trauma) results from the prolonged mechanical compression of muscle tissue, which leads to the breakdown of muscle cells and systemic release of intracellular contents [85]. It accounts for approximately 40% of rhabdomyolysis cases [86]. The pathogenesis of crush syndrome begins with ATP depletion, leading to energy failure within myocytes. This energy deficit leads to electrolyte imbalances, resulting from increased calcium influx into cells, osmotic water influx, activation of enzymes, and oxidative stress, ultimately culminating in cell death and the release of intracellular contents into the bloodstream [87]. In patients with crush syndrome, elevated levels of muscle-derived substances such as lactate dehydrogenase (LDH), aspartate aminotransferase (AST), creatine kinase (CK), myoglobin (Mb), and electrolytes were found [88].

Hypoxia, another cause of rhabdomyolysis in trauma patients, can result from vascular injury and increased pressure due to compartment syndrome, both of which impair blood flow and oxygen delivery [89,90].

Ischemia–reperfusion injury occurs when blood flow is restored to previously ischemic muscle tissue, triggering oxidative stress and inflammatory responses that exacerbate muscle cell necrosis. The initial ischemic insult results in oxygen and nutrient deprivation, compelling myocytes to switch to anaerobic metabolism, which leads to the accumulation of metabolic byproducts. Upon reperfusion, reintroducing oxygenated blood to the ischemic tissue induces the generation of ROS, massive calcium accumulation in ischemic muscles, and neutrophil infiltration into reperfused vessels, triggering an inflammatory cascade. These processes amplify cellular damage through necrotic and apoptotic pathways while initiating systemic inflammatory responses that can exacerbate tissue damage beyond the initial ischemic zone [86].

The divergent pathophysiological processes underlying rhabdomyolysis caused by crush syndrome and ischemia–reperfusion injury require distinct clinical management strategies. Further, interventions are necessary to restore tissue perfusion, mitigate oxidative stress, and modulate the inflammatory response.

Patients with TRR are at risk of developing severe complications, including cardiac failure, AKI, systemic inflammation, sepsis, hypovolemic shock, hyperkalemia, compartment syndrome, metabolic acidosis, and disseminated intravascular coagulation (DIC) [91,92].

#### Rhabdomyolysis-Associated Acute Kidney Injury: Pathophysiological Insights and Systemic Consequences

The emergence of AKI in rhabdomyolysis involves a combination of intrarenal vasoconstriction, direct tubular damage, ischemic injury, and tubular obstruction [93]. Myoglobin builds up and precipitates within the renal tubules, particularly under conditions of dehydration and vasoconstriction, while acidic urine exacerbates these events [93]. Precipitation predominantly occurs in the distal tubules, whereas proximal tubules are subject to direct cytotoxic insults [94,95]. Myoglobin nephrotoxicity intensifies in acidic environments, where unchecked production of ROS accelerates tissue damage [95,96].

Renal vasoconstriction is a defining feature of rhabdomyolysis-associated AKI, initiated by several mechanisms. Fluid depletion resulting from muscle injury leads to intravascular volume contraction, which activates the renin–angiotensin system and sympathetic pathways. Concurrently, vasoconstrictive agents, including endothelin-1, thromboxane A2, and tumor necrosis factor-α, as well as reduced NO bioavailability due to myoglobin binding, further diminish renal blood flow [97]. These vascular alterations are worsened by local oxidative damage and inflammation, which are characteristics of AKI secondary to endothelial dysfunction. Mouse models reveal that endothelin-1 (ET-1)-driven vasoconstriction contributes significantly to rhabdomyolysis-related AKI, underscoring the potential of targeting ET-1-mediated renal vasoregulation as a therapeutic strategy [98,99]. Additionally, heme-activated platelets released from necrotic muscle cells can induce AKI during rhabdomyolysis [100].

Ferroptosis—recently identified as an iron-dependent cell death pathway distinguished by intracellular iron overload, glutathione depletion, and lipid peroxidation—has likewise been implicated in rhabdomyolysis-induced AKI. Excessive myoglobin filtration fosters iron accumulation and oxidative membrane damage, hallmark features of ferroptosis, thereby making ferroptosis a viable therapeutic target in rhabdomyolysis [101].

A study performed in a trauma intensive care unit showed that 85% of critically injured patients exhibited biochemical signs of rhabdomyolysis, as indicated by elevated CK levels. Nonetheless, only 10% progressed to renal failure, and a mere 5% required RRT [102]. More recent evidence suggests that 40–50% of patients diagnosed with crush syndrome develop AKI, with 20% necessitating dialysis [103,104]. The mortality risk linked to rhabdomyolysis is mainly attributable to AKI, as reflected by higher mortality rates among rhabdomyolysis patients with AKI (24.8%) compared to those without AKI (11.8%) [105,106,107]. In trauma-induced rhabdomyolysis, the surge of myoglobin and potassium provokes systemic complications, notably acute renal failure and cardiac arrhythmias [108].

In TRR, heightened CK levels are closely tied to renal insufficiency; specifically, CK concentrations exceeding 5000 U/L display an independent association with AKI development [102,109,110,111]. Serum CK typically begins to rise within 2 to 12 h after muscle injury, peaks at 24 to 72 h, and subsequently declines at approximately 40% per day relative to the preceding day’s value [112]. In cases complicated by compartment syndrome, additional muscle injury may produce a “second-wave phenomenon,” evidenced by persistently elevated or rebound CK levels within 48 to 72 h of the initial trauma [89].

Although myoglobin is widely recognized as the principal pathogenic entity in rhabdomyolysis-induced AKI, it is infrequently quantified in clinical practice owing to its swift and variable metabolism—primarily outside the kidney—which hampers its diagnostic reliability [113,114]. Recent findings advocate for classifying rhabdomyolysis based on elevated serum myoglobin thresholds, ranging from 368 µg/L [115] to 3865 µg/L [116], as these measures more accurately predict AKI onset [103]. The wide range of serum myoglobin thresholds for predicting AKI in rhabdomyolysis—such as 368 µg/L and 3865 µg/L—reflects differences in patient populations, timing of measurement, AKI definitions, and assay methods. Lower thresholds may identify early risk, while higher peaks better correlate with severe outcomes in more extensive muscle injury.

Clinically, individuals with rhabdomyolysis frequently exhibit muscle weakness, pain, localized swelling, and dark or reddish urine. Pigmented casts in the urine and reddish-brown urine are standard. The ensuing AKI tends to develop more swiftly and with a sharper rise in plasma creatinine than other AKI forms, often featuring a reduced blood urea nitrogen (BUN)-to-creatinine ratio and more frequent oliguria than anuria. Notably, rhabdomyolysis-induced AKI demonstrates a low fractional excretion of sodium, reflecting pronounced preglomerular vasoconstriction and tubular blockage, rather than the enhanced urinary sodium typically observed in ischemic or toxic acute tubular necrosis [117].

Hyperkalemia, a serious complication in rhabdomyolysis, originates from tissue destruction, acidosis, hemolysis of transfused erythrocytes, and reduced potassium excretion due to renal failure. Immediate intervention is crucial to prevent fatal cardiac arrhythmias linked to elevated serum potassium. Following traumatic injury, patients often present with early hypocalcemia—attributable to calcium sequestration in damaged muscle tissue—and a subsequent phase of hypercalcemia. The initial decline in serum calcium is further aggravated by lower calcitriol synthesis and hyperphosphatemia. Furthermore, hypercalcemia emerges later when previously precipitated calcium is mobilized, parathyroid hormone sensitivity is restored, and vitamin D production resumes [117,118]. The release of phosphorus from necrotic muscle exacerbates hyperphosphatemia, a condition intensified by renal insufficiency that impairs phosphate clearance.

Hypoalbuminemia is also prevalent in individuals with crush syndrome. Arteriolar and capillary vasodilation, compounded by reduced hydrostatic pressure and falling albumin levels, predisposes patients to subsequent complications. Routine abdominal and limb circumference measurements facilitate the early detection of these risks [119]. According to KDIGO guidelines, early aggressive fluid resuscitation with isotonic crystalloids (e.g., 0.9% saline) is critical to maintain urine output (≥200–300 mL/h), dilute nephrotoxic myoglobin, and restore renal perfusion. The routine use of mannitol, sodium bicarbonate, or diuretics should be avoided; bicarbonate should be reserved for severe acidosis (pH < 7.1) and mannitol for refractory oliguria after hydration. Electrolyte imbalances are recommended to be promptly corrected. RRT is indicated for refractory electrolyte imbalances, acidosis, or fluid overload [120]. Emerging therapies targeting ferroptosis (e.g., iron chelators) and early fasciotomy for compartment syndrome may reduce complications [121,122]. Current guidelines prioritize hydration and caution against unproven adjuncts [123].

### 3.2. Kidney–Organ Crosstalk in Trauma Patients in the ICU

In critically ill patients, the onset and progression of AKI are closely linked to dysfunction across multiple non-renal organ systems. While this overall effect may primarily reflect systemic conditions, such as shock or widespread inflammation, another plausible explanation involves organ crosstalk, whereby the failure of one organ directly contributes to the dysfunction of others.

#### 3.2.1. AKI Associated with Traumatic Brain Injury

AKI associated with traumatic brain injury (TBI) demonstrates this phenomenon and is among the most extensively studied forms of organ crosstalk. The incidence of AKI among patients with TBI ranges from 12% to 17.7% [19,20,124], with severe cases (stage 3 AKI) being less common, accounting for approximately 2.1% [125]. However, higher incidences of up to 39.3% have been reported in cohorts characterized by higher injury severity scores and comorbidities predisposing to renal dysfunction [19]. Independent risk factors identified for AKI development in TBI patients include pre-existing renal disease, insulin-dependent diabetes, hypernatremia, administration of osmotic therapy [126], and aminoglycoside antibiotic use [127]. AKI typically manifests early, with a median onset of day 2 post injury, and is associated with prolonged ICU and hospital stays, increased morbidity, higher mortality, and more significant long-term disability [20,125,126]. TBI pathophysiology comprises two distinct phases: a primary injury phase at the moment of impact resulting in direct neuronal damage, cerebral edema, hemorrhage, ischemia, or diffuse axonal injury, followed by a secondary injury phase occurring over hours to days, characterized by ischemic, inflammatory, immune-mediated, and cytotoxic processes that disrupt the blood–brain barrier and exacerbate neuronal injury [128]. This neuroinflammatory cascade affects the activation of resident glial cells, including microglia and astrocytes, which release pro-inflammatory cytokines such as IL-1β and tumor necrosis factor-alpha (TNF-α). These mediators activate the JAK2/STAT3 and TLR4/NF-κB signaling pathways in renal tubular cells, contributing to apoptosis and fibrotic changes [129]. Elevated IL-6 levels, frequently observed post TBI, correlate with renal tubular injury markers like neutrophil gelatinase-associated lipocalin (NGAL) and the subsequent development of AKI and multiple organ dysfunction [130,131]. Additionally, catecholamine waves following TBI, notably elevated epinephrine and norepinephrine, induce renal vasoconstriction and sodium retention, exacerbating renal ischemia and brain injury severity [132]. Interventions scheduled to mitigate secondary brain injury, such as hyper-osmolar therapy, aggressive fluid resuscitation, and vasoactive drugs, may inadvertently compromise renal perfusion and function [133].

The gut–brain axis, a bidirectional communication system integrating neural, hormonal, and immune signals, is significantly disrupted following TBI. Injury-induced intestinal barrier dysfunction facilitates the translocation of microbial toxins, exacerbating systemic and cerebral inflammation [134,135,136]. Alterations in gut microbiota composition following TBI decrease beneficial commensal species and enhance pathogenic strains, accompanied by impaired gastrointestinal motility due to dysfunction of the enteric nervous system [137,138]. This dysbiosis contributes to AKI progression via increased uremic toxin production [139], disruption of short-chain fatty acid (SCFA) homeostasis, and immune–endocrine signaling dysregulation [140,141]. Reduced SCFA levels, typically renoprotective, may exacerbate renal injury [142]. Consequently, TBI-induced gut barrier dysfunction promotes systemic inflammation and immune dysregulation, aggravating kidney injury. Restorative interventions, such as targeted probiotic therapy and nutritional modulation, have shown promise in rebalancing gut microbiota, enhancing epithelial barrier integrity, and attenuating inflammation. These strategies may reduce the risk of TBI-associated AKI, though randomized controlled trials are still needed to confirm their efficacy [143,144].

Notably, the brain–kidney axis is bidirectional, as AKI can also impact the brain. Experimental studies in murine models of ischemic AKI have revealed increased infiltration of brain macrophages, neuronal pyknosis, activation of glial cells, and elevated blood–brain barrier permeability, all of which contribute to neurological deterioration [145]. Thus, preserving renal function is integral to protecting neurological outcomes.

Early identification of TBI patients at elevated risk for AKI is essential. Proactive interventions—such as individualized blood pressure targets, minimizing exposure to nephrotoxic agents, advanced hemodynamic monitoring, cautious use of vasopressors, and judicious fluid resuscitation—are crucial in this context [133]. Additionally, the use of colloids and mannitol should be approached with caution due to their potential to worsen kidney injury [146,147]. An integrated, organ-protective approach is crucial in enhancing the renal and neurological outcomes of TBI patients.

#### 3.2.2. AKI Associated with Thoracic Trauma

Another emerging area of investigation is the complex crosstalk between thoracic trauma and AKI, which reflects the intricate interplay between the pulmonary, cardiovascular, and renal systems in the context of critical injury [109]. Thoracic trauma, a major driver of trauma-related morbidity and mortality, is implicated in approximately 25% of trauma deaths and affects up to 60% of patients with polytrauma. The mortality rates associated with such injuries range from 20% to 25%, underscoring the clinical severity and systemic implications of chest trauma [148,149]. It can be broadly categorized into penetrating and blunt injuries; while penetrating trauma disrupts tissue integrity directly, blunt trauma induces internal damage through mechanisms such as acceleration–deceleration forces or compression, often without visible tissue disruption [150,151,152]. Blunt thoracic trauma constitutes nearly 70% of all chest injuries and approximately 15% of global trauma cases [153,154]. Mortality in these patients often results from combined pulmonary and extrapulmonary complications. Among these, AKI represents a particularly severe consequence, arising through a complex interplay of hemodynamic, respiratory, inflammatory, and neurohormonal pathways [109,155,156]. A key mechanism involves massive hemothorax due to damage to intercostal or mediastinal vessels, leading to significant blood loss—sometimes up to 6 L—causing hypovolemia, circulatory collapse, and subsequent renal hypoperfusion, which is a critical driver of ischemic tubular injury [157,158]. Pulmonary injuries such as contusions or pneumothorax further compromise gas exchange, resulting in systemic hypoxemia that exacerbates the inherent vulnerability of the renal medulla to oxygen deprivation. In parallel, thoracic trauma induces a robust systemic inflammatory response characterized by the release of pro-inflammatory cytokines, ROS, and DAMPs, which propagate endothelial dysfunction, microvascular injury, and leukocyte-mediated damage in both pulmonary and renal tissues [159]. Mechanical ventilation, often necessary in patients with severe chest trauma, may contribute to renal dysfunction by increasing intrathoracic pressure, thereby reducing venous return and renal perfusion while also enhancing systemic inflammation and predisposing to multi-organ dysfunction syndrome (MODS) [160].

Additionally, the neurohormonal response—including activation of the sympathetic nervous system, the renin–angiotensin–aldosterone system (RAAS), and vasopressin secretion—leads to renal vasoconstriction, fluid retention, and worsening pulmonary congestion. Acid–base disturbances and electrolyte imbalances, notably hyperkalemia and metabolic acidosis associated with AKI, further impair respiratory function and increase the risk of arrhythmias. This bidirectional lung–kidney interaction highlights the intricate pathophysiology of AKI in thoracic trauma and emphasizes the importance of a multidisciplinary approach to managing these critically ill patients [161,162].

#### 3.2.3. AKI Associated with Abdominal Trauma

Abdominal trauma, whether due to blunt or penetrating mechanisms involving the abdominal wall, visceral organs, or vascular structures, remains a major contributor to trauma-related morbidity and mortality, with global fatality rates ranging from 1% to 20%, depending on injury severity and healthcare system capabilities [163,164,165,166,167,168,169]. Among intra-abdominal organs, the spleen, liver, and kidneys are the most commonly reported sites of injury [170,171,172]. AKI, in abdominal trauma, is mediated by a multifaceted interplay of hemodynamic, inflammatory, and mechanical mechanisms. Hemorrhagic shock and vascular disruption are primary drivers of renal hypoperfusion and ischemic tubular injury [173], while crush injuries may induce rhabdomyolysis and consequent myoglobin-induced nephrotoxicity [174]. Moreover, surgical interventions and aggressive fluid resuscitation may precipitate abdominal compartment syndrome, wherein elevated intra-abdominal pressure further compromises renal perfusion [175,176]. Ischemia–reperfusion injury during hemorrhage control or bowel manipulation exacerbates oxidative stress [177]. In parallel, the systemic inflammatory response triggered by trauma induces endothelial dysfunction, renal vasoconstriction, and injury to tubular epithelial cells, thereby exacerbating renal damage. Inflammatory mediators, including neutrophils, lymphocytes, and platelets, orchestrate this response. Hematologic indices like the neutrophil-to-lymphocyte ratio (NLR), platelet-to-lymphocyte ratio (PLR), and others, including the monocyte-to-lymphocyte ratio (MLR), systemic inflammatory index (SII), systemic inflammatory response index (SIRI), and aggregate inflammatory systemic index (AISI), have similarly been correlated with adverse renal outcomes in surgical and trauma settings [178,179,180,181]. Trauma-related gut barrier disruption permits bacterial translocation and releases LPS, which activates renal TLR4, promoting the production of chemokines (e.g., CXCL1) and neutrophil infiltration, thereby fueling systemic inflammation through a gut–kidney axis [182,183]. The pro-inflammatory cascade, characterized by elevated cytokines, leukocyte infiltration, and endothelial dysfunction, amplifies renal insult. Emergency laparotomy, often necessitated in unstable abdominal trauma patients, further increases AKI risk due to intraoperative hypotension, blood loss, and postoperative complications—especially in elderly patients or those with comorbidities like diabetes, hypertension, and coronary artery disease [163,164,165]. Therapeutic exposures, such as nephrotoxic drugs and contrast agents, may further worsen renal dysfunction. Together, these mechanisms reflect a dynamic and bidirectional crosstalk between abdominal injury and renal impairment, reinforcing the need for early risk stratification and kidney-protective strategies in the management of abdominal trauma patients [184].

To summarize, in critically ill trauma patients, AKI emerges not as an isolated renal event but as a result of dynamic and bidirectional crosstalk between the kidneys and other injured organs—particularly the brain, thorax, and abdomen. Traumatic brain injury triggers systemic inflammatory responses, neurohormonal activation, and gut dysbiosis, which compromise the blood–brain barrier and exacerbate renal dysfunction through cytokine storms, endothelial injury, and altered hemodynamics. Thoracic trauma, including pulmonary contusion and hemothorax, contributes to AKI through hypoxemia, massive blood loss, inflammatory mediator release, and impaired renal perfusion—effects often worsened by mechanical ventilation and neurohumoral dysregulation. Similarly, abdominal trauma leads to hemorrhagic shock, rhabdomyolysis, and ischemia–reperfusion injury, while gut barrier compromise and intra-abdominal hypertension further propagate renal insult. In all cases, organ dysfunction is mutually reinforcing: AKI can exacerbate pulmonary edema, impair neurological outcomes, and amplify systemic inflammation. Recognizing these inter-organ interactions is essential for guiding early interventions, organ-protective strategies, and integrated trauma care to mitigate AKI-related morbidity and mortality.

### 3.3. Postoperative AKI in Trauma Patients in the ICU

Another major contributor to the AKI burden in trauma patients is postoperative AKI (PO-AKI), a common complication following major surgery. It accounts for 18–47% of hospital-acquired AKI cases [185]. PO-AKI is defined as AKI that meets KDIGO criteria within 7 days after surgery [186]. In trauma settings, PO-AKI is particularly prevalent and is associated not only with immediate postoperative complications but also with long-term adverse outcomes, including the outcome of CKD, cardiovascular events, and increased mortality [187]. An extensive cohort study involving 10,518 patients without pre-existing CKD who underwent major surgery reported a 30-day mortality rate of 1.9% in patients without AKI, compared to a significantly higher rate of 31% among those who developed AKI [188,189]. The etiology of PO-AKI is commonly multifactorial, encompassing preoperative, intraoperative, and postoperative factors that collectively impair renal microcirculation, increase oxygen demand, and trigger inflammation—mechanisms that can negatively influence both prognosis and the risk of progression to CKD [187,190]. Risk factors for PO-AKI in trauma patients include preoperative variables such as older age; underlying conditions like CKD, diabetes mellitus, cirrhosis, hypertension, and cardiac failure; emergency surgery; and use of ACE inhibitors or angiotensin receptor blockers [191,192,193]. Avoiding nephrotoxic drugs, such as gentamicin used in surgical prophylaxis, remains essential in PO-AKI prevention [194]. Additionally, albuminuria and low serum albumin levels are recognized risk factors [195]. Intraoperative risks include hypovolemia from blood loss, systemic inflammation, increased intra-abdominal pressure, hypotension due to anesthetic-induced vasodilation or decreased cardiac output, ischemia–reperfusion injury, and direct renal ischemia [196,197]. The EPIS-AKI study, a prospective international observational trial, revealed that intraoperative vasopressor use and aminoglycoside administration were risk factors for PO-AKI [189]. Extensive observational studies and clinical trials have shown that intraoperative hypotension—especially when the mean arterial pressure (MAP) falls below 60–70 mmHg or drops more than 30% from baseline—is strongly linked to postoperative AKI [198,199]. Maintaining an MAP above 65 mmHg as part of a goal-directed strategy has been shown to reduce complications, including AKI, and shorten hospital stays [200]. Although urine output during surgery has often been considered a poor predictor of postoperative AKI, a retrospective observational study involving 5894 patients undergoing elective or emergency major noncardiac surgery found that intraoperative oliguria lasting more than 120 min was independently associated with a higher risk of developing postoperative AKI [201]. Postoperatively, factors such as ongoing hypovolemia, persistent hypotension, cardiac dysfunction, systemic inflammation, exposure to nephrotoxins, elevated intra-abdominal pressure, acute lung injury, mechanical ventilation, and urinary tract obstruction may contribute to PO-AKI [186,191,202]. Patients with PO-AKI who still fulfill the KDIGO criteria for AKI more than 7 days after surgery are categorized as having postoperative acute kidney disease, up until 90 days, at which point it is classified as CKD [186].

Prevention and treatment of PO-AKI in trauma patients relies mainly on identifying those at high baseline risk and minimizing exposure to nephrotoxic factors through careful monitoring. A key tool in this process is the Kidney Health Assessment (KHA), a structured clinical evaluation that helps assess current kidney function and uncover individual risk factors for AKI [186]. The KHA involves a detailed history, including drug, social, and travel history, as well as a focused physical exam assessing fluid status, heart failure, and infection [186]. The treatment of PO-AKI shares many features with the treatment of AKI in other settings [203]. The 2012 KDIGO guidelines recommend supportive care such as fluid management, blood pressure control, and avoiding nephrotoxins—for preventing and treating AKI [120].

### 3.4. Contrast-Induced AKI

Contrast-induced AKI (CI-AKI) is defined as an increase in sCr by ≥0.3 mg/dL within 48 h or ≥1.5 times baseline within 7 days after contrast exposure, or urine output < 0.5 mL/kg/h for at least 6 h, when alternative explanations or etiology for renal impairment has been excluded [120,204]. In many patients—particularly critically ill trauma cases—multiple overlapping factors such as shock, sepsis, and nephrotoxic medications complicate the ability to attribute AKI solely to contrast media. Consequently, recent guidelines and studies advocate for terms like contrast-associated acute kidney injury (CA-AKI) or post-contrast AKI (PC-AKI), which refer to AKI occurring within 7 days of contrast exposure without assuming a direct causal relationship [205,206,207]. For this reason, CI-AKI, which has largely replaced the historical term contrast induced nephropathy (CIN), is a subtype of CA-AKI. Because these concurrent insults make it challenging to isolate contrast media as the sole cause of AKI, many cases labeled as CI-AKI may be multifactorial or overlapping with contrast exposure. To this extent, older studies failed to exclude other causes of AKI, such as patient condition, procedure type, and other confounding factors, and lacked proper controls. As a result, the higher incidence of AKI reported as CIN may represent CA-AKI rather than true CI-AKI, leading to an overestimation of incidence in the literature [206,208]. This confounding can lead to an overestimation of the actual nephrotoxic risk of contrast agents in patients with con-CKD and CKD [209].

Trauma patients are at considerable risk of developing CI-AKI, with incidence rates ranging from 1.9% to 19.4%, depending on the definition of AKI and the characteristics of the study population [22,23,24,25,210]. In CI-AKI, creatinine levels peak at 3–5 days and return to near baseline within 1–3 weeks [205]. It results from a combination of renal ischemia and the direct cytotoxic effects of iodinated contrast media on tubular epithelial cells. Initially, contrast administration generates a transient increase in renal blood flow, followed by a sustained reduction, mainly affecting the medulla, thereby promoting regional hypoxia [211]. This hypoperfusion is further aggravated by vasoconstrictive mediators, such as angiotensin II and endothelin-1, along with a diminished availability of vasodilators, like nitric oxide [212,213]. Additionally, increased blood viscosity and erythrocyte aggregation contribute to microvascular congestion and oxygen delivery impairment [214].

Further exacerbating renal injury, contrast agents can induce dehydration and hyper-osmolar states, both of which intensify renal hypoperfusion and predispose to tubular ischemia [215]. On a cellular level, contrast agents can cause tubular epithelial cell necrosis [216], apoptosis, and oxidative damage due to the production of free radicals [217], as well as endothelial dysfunction [218]. In vitro studies using porcine proximal tubular cell lines have shown that contrast media impair mitochondrial function and suppress cell proliferation. Notably, these effects were partly reversible upon withdrawal of the contrast agent, indicating that timely intervention may mitigate cellular injury [219].

Trauma patients possess unique vulnerabilities that increase their risk for CI-AKI when compared to the general population. Trauma-specific conditions—such as hypovolemic shock, elevated lactate levels, rhabdomyolysis, and high injury severity scores—are independently associated with increased risk for CI-AKI [25]. Comorbidities such as CKD, diabetes mellitus, and hypertension further amplify this susceptibility [24,220,221]. In a large cohort of over 12,000 patients undergoing contrast-enhanced CT, the incidence of CA-AKI rose with increasing severity of CKD—9.9% in stage 3A, 14.3% in stage 3B, and approximately 20.5% in stages 4 and 5. Although the CA-AKI rates were similar in stages 4 and 5, the need for dialysis within 30 days was significantly higher in stage 5 (54.1% vs. 18%) [221]. The study distinguished between CIN, defined by a creatinine rise within 48–72 h, and CA-AKI, which allows for up to 7 days. Despite the broader CA-AKI window, most creatinine peaks occurred within 24–48 h, suggesting a limited practical difference between the definitions.

Acute physiological disorders expected in trauma, including systemic inflammation, oxidative stress, and impaired renal perfusion, create a milieu facilitative to kidney injury. The frequent and urgent need for contrast-enhanced imaging in trauma care settings leads to repeated exposure to nephrotoxic agents. However, according to Giles and colleagues, repeated contrast administration within the early ICU stay does not significantly contribute to the development of AKI in polytrauma patients [24]. At the same time, another study found that cumulative contrast medium exposure from repeated contrast administration is an independent risk factor for AKI, especially in patients with severe trauma, pre-existing kidney dysfunction, or diabetes mellitus [222]. These studies differ in how they define contrast exposure, either by the number of scans or cumulative contrast medium exposure. Repeated scans do not necessarily increase AKI risk if the total contrast dose remains low, but large cumulative doses, particularly in patients with severe injuries, can raise the risk.

Hyper-osmolar contrast agents are thought to increase nephrotoxic risk via mechanisms such as vasoconstriction and tubular apoptosis, especially in vulnerable patients. Although iso-osmolar agents are more viscous, they have been shown to reduce nephrotoxicity in high-risk populations [223]. In a cohort of 284 trauma patients, 14% developed CI-AKI; however, after adjusting for variables such as age, injury severity, and lactate levels, CI-AKI was not found to be an independent predictor of mortality or complications [25]. This may be attributable to the typically transient nature of CI-AKI and the efficacy of early interventions such as prompt fluid resuscitation and standardized critical care protocols routinely operated in major trauma centers. Additionally, the limited sample size may have constrained the statistical power of the analysis.

According to an extensive retrospective cohort study using data from the National Trauma Databank (2017–2018), the type of contrast-enhanced imaging influences the risk of AKI requiring hemodialysis. Compared to patients not receiving contrast, those undergoing contrast-enhanced computed tomography (CT) had a 1.49-fold increased risk of requiring hemodialysis. Angiographic procedures alone increased this risk by 4.33-fold, while the combination of CT and angiography presented the highest risk, increasing the likelihood of requiring dialysis by 5.35 times [224].

Early identification of high-risk individuals—particularly those with baseline renal impairment or hemodynamic instability—enables timely prophylactic measures. In the BITCOIN study of patients undergoing coronary angiography, a higher urinary NGAL/creatinine ratio predicted contrast-induced AKI, while KIM-1 and calprotectin did not show diagnostic value. Though not conducted in trauma patients, the findings suggest that NGAL/creatinine may help identify contrast-related injury in high-risk settings [225].

Prevention of CI-AKI in trauma patients hinges on a multifaceted approach. Key strategies include pre- and post-procedural hydration with isotonic saline (1–3 mL/kg/hr before/after contrast), minimizing contrast volume, using iso-osmolar contrast media, discontinuing nephrotoxic drugs (e.g., nonsteroidal anti-inflammatory drugs (NSAIDs)) pre procedure, and avoiding repeated contrast exposure in short intervals. Intravenous hydration is preferred over oral hydration for preventing CI-AKI because it allows for easier control and monitoring of the fluid delivery rate [205]. However, a study found that intravenous hydration was not associated with lower risks of CA-AKI, chronic dialysis at discharge, and in-hospital mortality in patients with an estimated glomerular filtration rate (eGFR) < 30 mL/min/1.73 m^2^ undergoing intravenous administration of contrast media [226].

Adjunctive pharmacological agents, such as N-acetylcysteine (NAC) and sodium bicarbonate, have previously been used to reduce oxidative stress and improve renal protection [227]. However, larger and more rigorous studies, such as the 2018 PRESERVE trial, did not find a significant protective effect of NAC and sodium bicarbonate in lowering the risk of CI-AKI [228]. Consequently, the 2018 ESUR guidelines no longer support the routine use of NAC or sodium bicarbonate for CI-AKI prevention. However, the 2012 KDIGO guidelines published prior to the PRESERVE trial suggest, with a grade D recommendation, that oral NAC may be used alongside intravenous isotonic crystalloids in CKD [120,228]. CI-AKI pathophysiology involves direct tubular toxicity and hemodynamic insults, not solely oxidative stress. NAC’s antioxidant mechanism is irrelevant to these dominant pathways [229]. A recent study indicates that oral NAC is poorly absorbed, resulting in insufficient blood levels to effectively reduce oxidative stress [230]. While intravenous NAC can temporarily dilate the renal arteries, this effect appears to be limited to healthy kidneys. It does not occur in patients with CKD G3, the group most at risk for CI-AKI. Furthermore, they found that neither oral nor IV NAC significantly boosts intracellular glutathione or enhances overall antioxidant capacity in the bloodstream, indicating that it lacks the biological impact needed for kidney protection.

Emerging studies suggest that nicorandil and ranolazine may protect the kidneys in patients with mild-to-moderate renal impairment, offering promising new options to prevent CI-AKI [231,232]. Additionally, adding trimetazidine to hydration has been shown to reduce the risk of CI-AKI in patients with renal insufficiency undergoing coronary angiography [233]. Statin therapy before angiography also significantly lowers the risk of CI-AKI, according to a meta-analysis of multiple randomized trials [234]. However, these novel therapeutic approaches remain investigational and have not been formally integrated into established clinical practice guidelines to date.

Supporting hemodynamic stability and addressing underlying causes, such as shock or multi-organ dysfunction, is crucial to provide sufficient renal perfusion. Close monitoring of renal function before and after contrast administration enables early detection and timely management of CI-AKI. When integrated with modern trauma care protocols, these strategies collectively aim to mitigate the incidence and severity of CI-AKI in this high-risk population.

### 3.5. Drug-Induced AKI

In the trauma ICU, 19–25% of critically ill trauma patients experience drug-induced AKI, a significant contributor to morbidity and mortality [235,236,237].

Drug-induced AKI is defined by KDIGO criteria (elevated creatinine or reduced urine output) in temporal association with nephrotoxic drug exposure, absence of other clear causes, and improvement after drug withdrawal. It results from direct toxicity, hemodynamic changes, or immune reactions and may progress to acute kidney disease (AKD) or CKD if unresolved [120,238].

In pediatric populations, drug-induced nephrotoxicity occurs at a rate of around 16% [239], while elderly patients have an even higher incidence, reaching up to 66% [240]. According to a systematic review and meta-analysis, each nephrotoxic medication administered significantly increases AKI risk, with a 53% greater likelihood for each additional drug given (odds ratio: 1.53; confidence interval: 1.09–2.14) [241]. Given the complexity of trauma ICU care, where polypharmacy is often unavoidable due to the need for sedation, infection control, hemodynamic support, and analgesia, this finding highlights a key management challenge rather than a preventable error. It highlights the importance of nephrotoxin stewardship and routine monitoring of renal function.

The outcomes of drug-induced AKI can be severe, with in-hospital mortality rates averaging around 25% and dialysis dependency reaching 20% post injury [242]. Furthermore, a well-established connection exists between AKI and subsequent CKD, as demonstrated across diverse patient populations [243,244,245,246]. Longitudinal follow-up studies reveal that many patients, particularly children, who experience drug-induced AKI later portray markers of chronic kidney damage, such as a reduced GFR, hyperfiltration, proteinuria, and hypertension [239,243].

Understanding the detailed pathophysiologic mechanisms by which drugs cause kidney injury is critical for early recognition, prevention, and management of AKI, particularly in trauma ICU settings where polypharmacy and hemodynamic instability are common [247]. Drug-induced AKI can be extensively classified into three mechanisms: hemodynamic-mediated injury, direct tubular toxicity, and acute interstitial nephritis (AIN). Hemodynamic-mediated AKI often results from drugs that alter renal perfusion. For example, NSAIDs inhibit cyclooxygenase enzymes, thereby reducing vasodilatory prostaglandins such as PGE2 and prostacyclin, which leads to afferent arteriole constriction and a decrease in the GFR [248]. This effect is especially harmful in hypovolemic or hypotensive trauma patients. Similarly, angiotensin-converting enzyme inhibitors (ACEis) and angiotensin receptor blockers (ARBs) decrease the GFR by preventing angiotensin II-mediated constriction of the efferent arterioles. This mechanism can worsen renal function under trauma-related hypoperfusion. Direct tubular toxicity is another major pathway of drug-induced AKI in the ICU [247,249,250]. Aminoglycosides, such as gentamicin and amikacin, accumulate in proximal tubular cells through endocytosis, leading to mitochondrial dysfunction and oxidative stress, which in turn cause apoptosis and necrosis [251,252]. Vancomycin also exhibits nephrotoxic effects through the production of reactive oxygen species and the formation of obstructive tubular casts, particularly when co-administered with piperacillin–tazobactam. Polymyxins and colistin compromise the membrane integrity of tubular epithelial cells, leading to electrolyte imbalance, edema, and necrosis [253]. Amphotericin B induces renal injury through both direct membrane toxicity and vasoconstriction mediated by thromboxane, though liposomal formulations and hydration reduce its nephrotoxic potential. AIN, on the other hand, represents an immunologic mechanism [254]. β-lactam antibiotics, such as penicillins and cephalosporins, induce T-cell-mediated inflammation through cytokine activation, leading to interstitial edema and tubular dysfunction, often accompanied by allergic symptoms [251]. NSAIDs and proton pump inhibitors (PPIs) can cause delayed hypersensitivity reactions weeks to months after starting, often with subtle or no symptoms. This delayed onset is important in trauma ICU patients with prolonged stays or prior PPI use, where unexplained renal dysfunction may indicate PPI nephrotoxicity, and muted clinical signs, leading to delayed diagnosis [255,256].

The critical role of nephrotoxic medications, including antimicrobials and antivirals, has led to the widespread endorsement of drug-induced AKI as an unavoidable consequence in modern medicine. However, decreasing unnecessary exposure, particularly to avoidable nephrotoxins such as NSAIDs, could significantly mitigate risks. A multi-center cohort study by Yasrebi-de Kom et al. explored the impact of nephrotoxic drugs on AKI development in adult ICU patients [257].

The study identified aminoglycosides and vancomycin as major contributors, both of which are associated with tubular toxicity, particularly when used in combination. NSAIDs impair renal perfusion by reducing prostaglandin synthesis, while diuretics, although essential for fluid management, can worsen hypovolemia and renal ischemia.

Intravenous contrast agents are a significant cause of contrast-induced nephropathy (CIN), particularly in patients with pre-existing kidney dysfunction.

Admitting the absence of robust pharmaco-epidemiological studies addressing the nephrotoxic risks of medications in critically ill patients, Gray et al. conducted a comprehensive assessment using expert consensus [258]. Their work aimed to prioritize and categorize 167 medications commonly administered in the ICU based on their potential to induce kidney injury. Drugs like aminoglycosides, antifungals, and immunosuppressants, already known for their kidney risks, were again found to be strongly associated with AKI. However, less expected were the findings around opioids, phosphodiesterase inhibitors, and certain sympathomimetics (those that act on both alpha- and beta-receptors), which also appeared to increase AKI risk, despite limited clinical data backing this up.

The reasons behind these associations may involve low blood pressure, buildup of active drug metabolites, or constriction of blood vessels in the kidneys. That said, there were also hints that some of these drugs might offer protective effects in specific contexts. The findings were inconsistent. In many cases, associations weakened or disappeared in secondary analyses, likely influenced by timing, hemodynamic status, or co-medication.

Some drugs that often spark debate around kidney risk, like iodinated contrast agents and proton pump inhibitors, did not show a significant link to AKI in this review. This lines up with previous studies [259]. NSAIDs (excluding aspirin) also did not show a strong connection, which contradicts long-held beliefs. This could be due to confounding by contraindication, where sicker patients who are more vulnerable to AKI are simply less likely to be prescribed these drugs. Interestingly, when researchers adjusted for additional variables, the risk estimate increased. This suggests that hidden patient factors, like chronic high blood pressure or smoking history, may be skewing the data.

In contrast, some drug classes, according to some studies, were tied to a lower risk of AKI. These included antiepileptics, ACE inhibitors, and glucose-lowering medications such as metformin. Metformin has been identified in recent studies as having potential kidney-protective effects, although the results were not consistent across all models [260,261]. In a multi-center cohort study, ACEis showed a protective effect in the primary analysis; however, this effect was attenuated in sensitivity testing due to residual confounding [257]. The use of ACEis and ARBs in these vulnerable patients needs caution, as these medications can worsen kidney function, especially when combined with other nephrotoxic drugs like NSAIDs and diuretics [262]. Research indicates that the use of ACEis and ARBs is associated with a higher risk of AKI in patients admitted for medical emergencies [263,264]. As a result, clinical practice recommendations commonly recommend discontinuing these medications in patients with AKI or other acute conditions due to conditions related to renal hemodynamics, a practice known as the “sick day rule” [265]. While ACEi/ARB therapy may offer benefits by reducing mortality in AKI patients, it also poses a risk of exacerbating AKD [266]. In alignment with expert consensus, the Acute Disease Quality Initiative (ADQI) and PeriOperative Quality Initiative recommend discontinuing ACEi/ARB therapy at least 24 h before surgery in high-risk patients, with reevaluation for reintroduction after 48 h if the patient stays stable and free from AKI [186]. Among antiepileptics, valproic acid stood out for consistently being associated with a lower risk of AKI across all models [267]. Lab studies indicate that it may help protect the kidneys during events such as trauma or blood loss. However, the mechanism of this advantage remains unclear, possibly due to improved blood flow or cellular mechanisms.

Overall, these patterns seem to reflect how drugs are used in real-world ICU care more than they do the drugs’ inherent toxicity. Practices like dose tweaking, close monitoring, and keeping hemodynamics stable likely help reduce the risk of drug-induced AKI [258]. Recent advances propose a more nuanced approach to classifying drug-induced AKI. The 23rd ADQI has introduced a biomarker-driven classification, categorizing AKI based on the presence or absence of kidney dysfunction and tissue damage [268]. The four categories are (1) neither dysfunction nor damage (pseudo AKI), typically associated with benign elevations in creatinine from drugs like cimetidine or cobicistat; (2) dysfunction without tissue damage, often resulting from hemodynamic effects of medications such as ACE inhibitors, ARBs, and Sodium–Glucose Cotransporter 2 inhibitors (SGLT2 inhibitors); (3) damage without apparent functional impairment, observed initially with drugs like aminoglycosides and vancomycin; and (4) both dysfunction and damage, characteristic of NSAIDs, calcineurin inhibitors, and amphotericin B. Urinary biomarkers such as KIM-1 enable early detection of proximal tubule injury and help differentiate tubular from glomerular damage, while NGAL and clusterin assist in identifying tubular involvement and distal tubule injury, respectively. Combining these with markers like cystatin C for overall kidney function and interleukin-18, beta-2 microglobulin, and alpha-glutathione S-transferase for specific injury patterns can improve diagnosis, monitoring, and assessment of drug-induced AKI in trauma patients [269].

To prevent drug-induced AKI, trauma ICU management should prioritize individualized dosing modified for renal function, incorporate therapeutic drug monitoring, and enforce strategies that support optimal renal perfusion, all while remaining vigilant about potential nephrotoxic drug interactions. Additionally, integrating urinary-biomarker-driven classification into clinical practice holds significant promise for enhancing diagnostic accuracy, guiding targeted therapeutic interventions, monitoring treatment response, and ultimately improving patient outcomes in the trauma ICU setting.

## 4. Emerging Biomarkers for Early Detection and Prognosis of Trauma-Related Acute Kidney Injury

### 4.1. Beyond Creatinine: Biomarker Insights into Early AKI in Trauma

Early AKI diagnosis remains challenging due to the reliance on sCr levels and changes in urine output—both of which lack sensitivity and specificity for renal injury. Serum Cr is a delayed indicator, often taking 2–3 days to rise following renal insult, and it typically only increases after more than half of the kidney’s functional reserve is lost [270]. Fluid status can further confound creatinine levels, masking or mimicking AKI when intravascular volume fluctuates. Reduced creatinine synthesis—particularly in children, older individuals, or those with low muscle mass—also complicates accurate assessment [271]. Likewise, sCr cannot distinguish between functional and structural kidney injuries, nor can it specify whether the damage is localized to the glomeruli or tubules, thereby limiting its diagnostic precision [272]. Even when sCr returns to baseline, significant kidney damage, such as fibrosis and abnormal gene expression, can persist, underscoring creatinine’s limited ability to reflect long-term renal outcomes [273,274] accurately.

Cystatin C is another well-known biomarker included in the assessment of chronic kidney failure. It is a low-molecular-weight protein created by all nucleated cells, freely filtered at the glomerulus, and almost completely reabsorbed and catabolized by proximal tubular cells without tubular secretion. Its serum concentration, therefore, reflects the GFR more directly than creatinine and is far less affected by muscle mass, age, or sex—qualities that confer superior sensitivity for early kidney dysfunction in critically ill patients. However, data in trauma cohorts remain limited [275].

Several studies have evaluated its performance as a GFR marker, and some recommend combining cystatin C with creatinine to optimize accuracy [276,277].

In a meta-analysis of 24 cohorts, abnormal cystatin C levels were defined as being between 0.9 and 1.4 mg/L to detect renal impairment; however, Asian populations may require adjusted equations due to systematic bias [278]. A serum cystatin C level above 0.78 mg/L on day one, as identified by Zand et al. in trauma ICU patients, was a strong independent predictor of AKI within the first week (OR 6.14; 95% CI 2.5–14.7; *p* < 0.001) [279,280]. Nonetheless, serum cystatin C can still be influenced by demographic factors such as ethnicity and body composition, and existing cystatin C-based GFR equations lack validation in the critically ill, underscoring the need for ICU-specific formulas [280]. Additionally, serial cystatin C measurements have not been shown to outperform creatinine or eGFR for AKI diagnosis in the emergency department; however, a single elevated cystatin C measurement at admission—alone or in combination with creatinine and the eGFR—predicts a higher risk of AKI [281].

Despite their broad use, creatinine and cystatin C primarily reflect alterations in glomerular filtration and often rise only after significant injury has occurred, hampering early detection [5,6,7]. In contrast, modern injury biomarkers—such as NGAL, KIM-1, IL-18, and liver-type fatty acid-binding protein (L-FABP)—are released directly from damaged tubular cells, allowing detection of acute insults within hours.

NGAL, a molecule released by activated neutrophils and damaged tubular epithelial cells, has been investigated in relation to AKI. NGAL is expressed in various tissue-specific isoforms and may function as a growth factor in multiple tissues [282]. Mishra et al. demonstrated increased NGAL expression in renal tissue following ischemic injury, accompanied by elevated plasma and urine NGAL levels [283]. Increased levels demonstrated significant diagnostic and prognostic utility, particularly in trauma-related contexts where ischemic and nephrotoxic insults occur [284,285,286]. Frelich et al. found that NGAL levels at admission, along with other biomarkers such as HMGB-1, creatinine, and myoglobin, were predictive of AKI in severely injured patients. However, in those with urinary tract trauma, NGAL did not particularly differ between patients who developed AKI and those who did not, suggesting its limited reliability in this subgroup [287,288].

The urinary NGAL level reliably predicts AKI severity and the necessity for renal replacement therapy. However, its performance may be influenced by systemic inflammation, comorbid conditions, and urinary tract injury, common in trauma populations [288,289].

KIM-1 is virtually undetectable in healthy kidneys because proximal tubular epithelial cells express it at only trace levels. Ischemic insult, ischemia–reperfusion injury, and exposure to myoglobin or to nephrotoxic drugs trigger rapid upregulation and shedding of KIM-1 into urine and plasma, making its surge a sensitive indicator of proximal tubule injury. Numerous studies have demonstrated that urinary KIM-1 reliably predicts persistent AKI and CI-AKI with high sensitivity and specificity (e.g., area under the curve > 0.80). However, baseline elevations in CKD can reduce its specificity [290,291,292]. Despite promising results in other clinical settings, data on the predictive value of KIM-1 for AKI in trauma patients remain scarce, highlighting the need for further research in this specific population.

Additionally, inflammatory biomarkers such as IL-18 and MCP-1 mirror systemic inflammation and immune activation, which are frequently present in trauma-associated sepsis. Urinary IL-18 notably improves early AKI detection in septic patients, a common complication in severe trauma, enhancing clinical decision-making and potentially guiding early intervention [293,294,295].

Additionally, the pro-inflammatory chemokine C-C motif chemokine ligand 14 (CCL14) has shown strong predictive capability in patients with established AKI, particularly in predicting disease progression [296]. The RUBY trial confirmed that elevated CCL14 concentrations were associated with persistent AKI and delayed renal recovery in critically ill patients [297]. Koyner et al. determined that a urinary CCL14 cutoff of 1.3 ng/mL or higher accurately identified patients at risk of persistent AKI or subsequent adverse outcomes such as the need for RRT [298]. An ongoing study (NCT05275218) on surgical patients is assessing the impact of CCL14-guided supportive measures on renal outcomes. Further research is needed to assess the utility of CCL14 as a biomarker for AKI prediction, specifically in the trauma patient population.

DKK3, a protein involved in the wingless-related integration site (Wnt)/β-catenin signaling pathway, has emerged as another promising biomarker. It is a stress-responsive glycoprotein secreted by renal tubular epithelial cells that drives tubulointerstitial fibrosis, which can also signal vulnerability to acute damage. Schunk et al. revealed that preoperative urinary DKK3 levels in patients undergoing cardiac surgery reliably predicted postoperative AKI and correlated with long-term renal dysfunction [299]. Similarly, a study by Yao Sun et al. in ICU patients after noncardiac surgery found that high serum DKK3 levels were significantly associated with an increased incidence of AKI and adverse outcomes, including AKI or death [300]. Additionally, DKK3 was reported as an independent predictor of CI-AKI even in the absence of overt CKD [301]. There are no studies to date evaluating DKK3 as a predictor of AKI specifically in trauma patients. Further research is required to assess the utility of DKK3 in predicting AKI specifically within the trauma patient population.

The routine measurement of such predictive biomarkers, such as NGAL, CCL14, and DKK3, could help clinicians identify long-term renal risks, allowing for more informed decision-making regarding the timing of elective procedures and other critical interventions.

L-FABP is a 14 kDa cytoplasmic transporter that shuttles reabsorbed free fatty acids from the proximal tubular lumen to mitochondria and peroxisomes, thereby sustaining β-oxidation and cellular energetics under physiological conditions. When renal perfusion falls, whether due to hemorrhage in major trauma, cardiopulmonary bypass, sepsis, or contrast exposure, tubular hypoxia and the attendant surge in reactive oxygen species convert fatty acids into cytotoxic lipid peroxides. This oxidative stress transcriptionally upregulates L-FABP, driving its rapid appearance in urine [302,303,304]. Several perioperative and critical care studies show that urinary concentrations rise within 4–6 h of the inciting insult, preceding creatinine-based AKI diagnoses by a full day and correlating with both injury severity and longer-term outcomes [305,306]. A practical, semi-quantitative bedside immunoassay allows clinicians to classify results as negative, weak positive, or strong positive: in trauma cohorts, a positive test at 6 h signals ongoing tubular ischemia, while conversion from weak positive at 6 h to negative at 12 h suggests reperfusion; persistent positivity at 12 h identifies patients at the highest risk for evolving AKI. Because L-FABP tracks dynamic changes in renal blood flow, repeating the assay after initial resuscitation can help clinicians determine whether hemodynamic optimization is sufficient or whether nephrotoxic “second hits” still threaten kidney viability [307]. In trauma patients, urinary L-FABP effectively reflects real-time kidney tubular ischemia and reperfusion, making it a valuable early biomarker for AKI. A prospective study of 100 ICU trauma patients showed that elevated urinary L-FABP at 6 and 12 h post injury was strongly associated with AKI development, even after adjusting for injury severity and other factors, highlighting its potential for early AKI prediction and timely intervention [308].

Emerging biomarkers, such as soluble urokinase plasminogen activator receptor (suPAR) and chitinase-3-like protein 1 (CHI3L1, also known as YKL-40), further enhance the AKI biomarker landscape, providing prognostic insights that are particularly relevant in complex trauma scenarios, including sepsis and inflammatory complications [309,310,311,312]. suPAR is evaluated as a promising biomarker for predicting postoperative AKI, with higher preoperative levels strongly associated with both increased incidence and severity of AKI. Its predictive value may enable earlier identification of high-risk patients and guide preventive perioperative strategies [310].

Biomarkers for AKI risk stratification have been approved by the U.S. Food and Drug Administration (FDA) since 2012 and have been incorporated into recent guideline recommendations, particularly in the setting of cardiac surgery [313,314].

One key development is the measurement of urinary tissue inhibitor of metalloproteinase-2 (TIMP-2) and insulin-like growth factor-binding protein 7 (IGFBP-7) under the NephroCheck™ (NC) assay, which has shown considerable promise for early AKI detection after cardiac surgery [314]. Identified as markers of cellular stress and repair, they provide critical insights into cell cycle arrest mechanisms initiated by renal ischemia, ischemia–reperfusion injury, inflammation, or nephrotoxicity—conditions frequently seen in polytrauma patients. Building on this foundation, the BRAVA study recently extended these findings to the emergency department, showing that NephroCheck™ effectively predicts not only AKI development but also short-term mortality in high-risk ED patients with diverse conditions, including sepsis, acute coronary syndrome, trauma, shock, major bleeding, and decompensated organ failures (e.g., heart, liver). This underscores its broader applicability in dynamic clinical settings where rapid risk stratification is critical [315].

A recent study evaluating the NephroCheck™ test in trauma patients investigated the clinical utility of TIMP-2 and IGFBP-7 in identifying early AKI [16]. The findings indicated that individuals who evolved early AKI were commonly older and had a higher prevalence of cardiac comorbidities. Higher biomarker levels were also associated with the need for RRT and episodes of hypotension. Multivariable analysis demonstrated that age, pre-existing cardiac disease, and TIMP-2 × IGFBP-7 values were the most significant predictors of AKI, yielding an area under the curve (AUC) of 0.792. Another study found that in ICU patients those with trauma who had elevated TIMP-2 × IGFBP7 levels at ICU admission were significantly associated with the development of AKI within 7 days [316].

These observations suggest that TIMP-2 × IGFBP-7 measurements may be an effective tool for identifying trauma patients at raised risk for clinically significant tubular damage and AKI.

In another study, Faust et al. examined the plasma levels of mtDNA, nuclear DNA (nDNA), and nucleosomes, reporting a strong correlation between these markers and the development of AKI in trauma patients [317]. Traumatic injury patients who eventually manifested AKI exhibited higher admission levels of these biomarkers compared to those who did not develop AKI. Crucially, this was the first study to propose that nDNA levels at presentation might serve as an early indicator of AKI and potentially respond to targeted therapeutic strategies in trauma populations.

IM Schmidt et al. identified a panel of novel, validated plasma biomarkers of acute tubular injury (ATI), which may serve as non-invasive diagnostic tools and potential therapeutic targets in AKI and related kidney pathologies. The plasma proteomic signatures of ATI were analyzed using data from 434 individuals with biopsy-confirmed kidney disease (Boston Kidney Biopsy Cohort, BKBC) [318]. Plasma proteins were quantified using SOMAscan (6592 aptamers), and ATI severity was scored histologically. The goal was to identify circulating biomarkers associated with the severity of ATI. The validation was conducted with findings of three cohorts: the Kidney Precision Medicine Project (KPMP) assessed protein expression in plasma and kidney tissue (tubulointerstitial vs. glomerular) from individuals with AKI and healthy controls; the ARIC study (*n* = 4610) evaluated whether BKBC-identified biomarkers predicted incident AKI over 6.8 years; and the CHROME study (*n* = 268 ICU patients) tested biomarker associations with severe AKI within 7 days of admission.

The key findings included 156 plasma proteins significantly associated with ATI severity. Among the most strongly upregulated were osteopontin (SPP1), MRC-1, tenascin C (TNC), KIM-1, WFDC2 (HE4), and GDF-15. These proteins were also elevated in tubulointerstitial compartments (KPMP), associated with incident AKI (ARIC), and linked to severe AKI outcomes (CHROME). Pathway analysis highlighted immune system dysregulation and organelle stress responses (e.g., mitochondrial and endoplasmic reticulum stress) as central in ATI. Single-cell data confirmed the expression of SPP1, GDF-15, and WFDC2 in tubular epithelial cells [318]. This study was a serious attempt to systematically characterize plasma protein biomarkers associated with histologically confirmed ATI, validate their association with AKI outcomes in diverse patient populations, and reveal key molecular pathways (immune and cellular organelle stress) driving tubular injury.

In trauma patients, Gökmen Aktas et al. demonstrated that urinary proteomic biomarkers analyzed by capillary electrophoresis coupled with mass spectrometry could effectively predict AKI and mortality. By applying selected classifiers such as CKD273 and AKI204, this approach enabled early risk stratification and supported more personalized decisions concerning treatment and timing of intervention [319].

**Figure 2:**
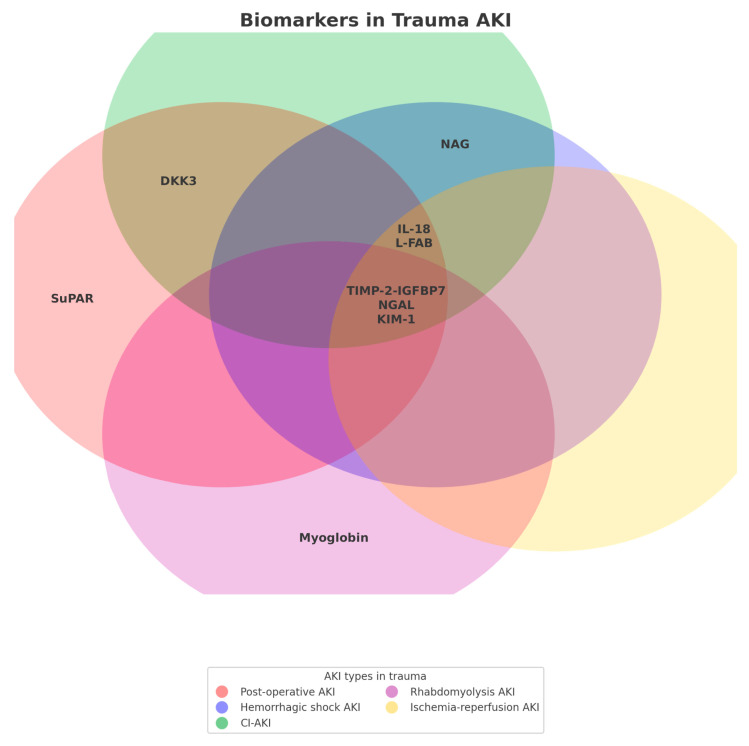
Mapping early diagnostic biomarkers across different TRAKI phenotypes.

To summarize, it should be noted that conventional surrogates of glomerular filtration, sCr, and, to a lesser extent, cystatin C, rise only after substantial nephron loss and are confounded by fluid shifts, muscle mass, and critical-illness physiology. Consequently, they miss the brief therapeutic window during which renal insults in major trauma might still be reversible. A new generation of structural and stress response biomarkers overcomes this lag by registering tubular hypoxia, inflammation, or cell cycle arrest within hours. Urinary or plasma NGAL, KIM-1, IL-18, L-FABP, and CCL14 effectively detect ischemic or nephrotoxic injury well before changes in creatinine occur. At the same time, FDA-cleared TIMP-2 × IGFBP-7 (NephroCheck) quantifies tubular cell cycle arrest and has already proven helpful in polytrauma cohorts. Biomarkers such as DKK3 and suPAR identify patients whose kidneys are primed for fibrosis and thus at heightened risk of both acute and chronic dysfunction. Proteomic classifiers, such as CKD273 and AKI204, further enhance early risk stratification by profiling urinary peptide signatures, thereby supporting personalized treatment strategies. When interpreted as a panel—rather than individually—these markers enable clinicians to distinguish between functional and structural AKI, assess the likelihood of progression to RRT or death, and titrate renoprotective interventions in real time. While ongoing studies aim to validate and optimize these biomarker panels specifically in trauma populations, integrating biomarker-guided risk stratification into trauma care pathways promises to shift AKI management from passive detection to proactive prevention [16,270,271,272,273,283,299]. Table 1 presents the main studied injury biomarkers in AKI in trauma settings, and Figure 2 shows the mapping of early diagnostic biomarkers across different TRAKI phenotypes.

At the center, shared by all five AKI subsets, were TIMP-2·IGFBP7, KIM-1, and NGAL. Biomarkers present in all categories except rhabdomyolysis AKI included L-FAB and IL-18. DKK3 was uniquely shared between postoperative AKI and contrast-induced AKI, while NAG was specific to the overlap of hemorrhagic shock AKI and contrast-induced AKI. Myoglobin was exclusively associated with rhabdomyolysis AKI, whereas suPAR appeared only in postoperative AKI.

### 4.2. Tracking TRAKI with Sequential Urinary Biomarkers (FENa, FEU, and FEK)

Recent investigations have raised questions about the traditional “pre-renal” paradigm, which links low urinary sodium levels to diminished renal perfusion, particularly in septic patients [203,320]. These studies observed that, in hyperdynamic sepsis, low urinary sodium concentrations can continue despite normal or increased renal blood flow (RBF), indicating that urine biochemistry may not reliably reflect a patient’s volume status and renal perfusion under these conditions. Furthermore, it has been reported that even in the early stages of tubular injury, patients often present with a “pre-renal” biochemical profile [321]. Specifically, biomarkers associated with tubular stress and injury are elevated, despite low fractional excretion of sodium (FENa) or urea (FEU), suggesting that urinary sodium retention may occur very early in the development of AKI.

Experimental sepsis models show decreased urinary sodium despite increased RBF. Maciel et al. proposed that low UNa reflects renal microcirculatory stress (RMS), independently of total RBF changes [322,323]. RMS is a pathological condition marked by impaired microvascular blood flow and oxygen delivery in the kidney’s cortex and medulla. This causes localized ischemia and cellular dysfunction, leading to early kidney injury—even when overall renal blood flow is normal or increased. RMS disrupts oxygen balance and promotes tubular cell damage, serving as a key mechanism in early AKI in critical illnesses like trauma and sepsis [34,324]. In their study of ICU patients with sepsis, trauma, and postoperative complications, they found that both transient and persistent AKI groups exhibited a precipitous decline in UNa one to two days prior to the clinical diagnosis of AKI, accompanied by a marked rise in the fractional excretion of potassium (FEK). Subsequently, UNa returned to normal in transient AKI but remained low in persistent AKI (Table 2). As AKI progressed, the UNa levels varied according to the GFR, the severity of tubular injury, impaired sodium reabsorption, and sodium backleak [325]. Notably, the FENa showed no significant changes between or within the groups and remained around 0.5%, even during this period [323]. This pattern persisted irrespective of diuretic usage. These findings suggest that diminished glomerular sodium filtration, rather than increased tubular sodium reabsorption, predominantly contributes to the early decrease in UNa. As a result, the FENa alone may not be a reliable early indicator of AKI, particularly in surgical populations [326].

In critically ill patients without AKI, the FENa is typically low, making additional reductions hard to detect in the early phases of AKI [323]. By contrast, sequential monitoring of UNa can magnify these subtle shifts in the FENa—even a slight drop in the FENa may precipitate a marked decrease in UNa. Consequently, UNa tracking can offer greater sensitivity for detecting incipient AKI in critically ill patients. Morais et al. similarly found that ICU patients who eventually developed AKI displayed lower urinary sodium excretion at least one day before the diagnosis, potentially reflecting elevated urinary sodium transporter levels [327]. Urine chemistry profiles may also vary in the severity and timing of AKI. In milder forms of renal insult, low urinary sodium excretion may anticipate impending AKI and coincide with higher sodium transporter expression. Conversely, in more severe injury, impaired tubular function can result in diminished solute reabsorption.

Previously, a study involving transplanted patients noted that a reduction in the urinary sodium concentration correlated with early stages of graft rejection [328], lending further support to low urinary sodium excretion as an early marker of renal compromise. Extending these observations, Maciel et al. propose that RMS is a unifying pathway for the onset of early AKI, regardless of the inciting etiology, especially in critically ill patients. Within this framework, any factor precipitating sodium retention may signify RMS, offering a more accurate descriptor than the traditional notion of “pre-renal” AKI [325].

Since urea transport functions independently of sodium transport mechanisms, the FEU has been deemed more reliable than the FENa in patients receiving diuretics. Based on the available evidence, the KDIGO guidelines recommend using an FEU threshold of 35% to help determine the etiology of AKI, especially in individuals on diuretic therapy [120]. Observational studies involving adult cardiac surgery and ICU cohorts have further explored the FEU for the early detection of AKI [326,329]. In these investigations, the FEU demonstrated diagnostic performance comparable to that of NGAL; notably, a cutoff value below 40% effectively distinguished transient from persistent AKI in ICU patients, even those on diuretics.

However, other studies have concluded that the FEU fails to differentiate transient from persistent AKI reliably and does not consistently predict AKI in critically ill patients [330,331]. Recent evidence also challenges the notion that loop diuretics have little effect on the FEU; instead, loop diuretics can significantly alter the FEU, and the timing of administration, as well as individual patient response, may substantially affect FEU readings and potentially shift AKI classification [332]. Moreover, the FEU appears to be less reliable in older adults and septic patients, as factors such as endotoxemia and aging reduce the expression of renal urea transporters, potentially leading to elevated FEU, even under hypovolemic conditions [333,334]. Consequently, an FEU exceeding 40% in these populations may not be indicative of early AKI, making it unsuitable for trauma patients in the ICU. Consistent with these findings, Maciel et al. reported that although the FEU decreased from two days before to one day before AKI diagnosis in both transient and persistent AKI, there were no significant differences between AKI and non-AKI groups, rendering it ineffective for early AKI detection [323].

In contrast, recent research highlights the potential of urinary potassium excretion as a predictor of AKI. Studies have indicated that urinary potassium levels correlate with creatinine clearance and may serve as a straightforward and accessible indicator of AKI risk in ICU settings [335,336] (Table 2). Maciel et al. demonstrated that the FEK rose in both transient and persistent AKI during the two days preceding AKI onset, then returned to normal in transient AKI but remained elevated in persistent AKI [323]. Diuretic use did not substantially alter FEK trends, and patients with persistent AKI presented significantly higher FEK values from day 0 to day 2. Further investigation in cardiac surgery patients corroborated the utility of FEK monitoring in evaluating AKI severity in critically ill individuals [337].

In trauma ICU patients, sequential monitoring of urinary sodium and potassium excretion can provide early warning of renal microcirculatory stress and imminent AKI. Detecting these changes promptly allows clinicians to optimize fluid resuscitation, avoid nephrotoxic exposures, and implement early nephroprotective strategies—potentially reducing the risk of persistent kidney injury and improving recovery outcomes in this vulnerable population. A summary of the roles of sequential urinary electrolyte biomarkers (FENa, FEU, and FEK) is presented in Table 2.

**Table 2:** The roles of sequential functional urinary biomarkers, FENa, FEU, and FEK, in early detection and etiologic differentiation of acute kidney injury in critically ill patients.

Biomarker	Mechanism	Key Observations	Strengths	Limitations	References
Urinary Sodium (UNa)	Reflects **sodium handling** by the kidney. Early drop in UNa can indicate **renal microcirculatory stress (RMS)** rather than just “pre-renal” hypoperfusion.	-Can be **low** even with normal or ↑ renal blood flow (e.g., sepsis). -**Declines ~1–2 days** before AKI diagnosis in both transient and persistent AKI. -Recovers if AKI is transient; remains low if AKI persists. -Not significantly altered by diuretic use.	-**Sensitive** for early AKI detection. -Magnifies subtle changes in FENa.	-Influenced by GFR changes, tubular damage severity, and backleak. -No universal threshold validated across all populations.	[203,320,321,322,323,325]
Fractional Excretion of Sodium (FENa)	Measures the **proportion of filtered sodium excreted**. Often used to distinguish “pre-renal” from intrinsic AKI but can be misleading in critically ill or diuretic-treated patients.	-Frequently ~0.5% in ICU patients, showing **little change** even with early AKI. -May be **unreliable** under diuretic use, high-output states, or sepsis.	-Simple, established index in stable, non-ICU settings.	-**Insensitive** for early AKI in critically ill patients. -Heavily confounded by diuretics and hyperdynamic circulation.	[203,320,321,326]
Fractional Excretion of Urea (FEU)	Based on **urea transport**, relatively independent of sodium transport, and proposed to be more reliable in diuretic therapy.	-KDIGO suggests FEU < 35% to evaluate AKI etiology in diuretic users. -Some studies: FEU < 40% distinguishes transient vs. persistent AKI. -Other studies: **no consistent predictive value** in older adults or sepsis. -Loop diuretics can **significantly affect** FEU.	-May be helpful in patients on diuretics where FENa is misleading.	-**Confounded** by loop diuretics, age, and sepsis (altered urea transport). -Not consistently predictive across diverse ICU populations.	[322,326,329,330,332]
Fractional Excretion of Potassium (FEK)	Reflects renal **potassium handling**. Rising FEK may signal early **tubular stress/injury**.	-**Increases 1–2 days** prior to AKI diagnosis; **remains high** if AKI persists or returns to normal if transient. -**Unaffected** or minimally influenced by diuretic use. -Correlates with creatinine clearance in ICU patients.	-**Early marker** of tubular injury. -Less confounded by diuretics.	-Requires more large-scale validation. -May be affected by other electrolyte or acid–base disturbances.	[323,335,336,337]

The following biomarkers are considered:

- UNa: This is emphasized as a potentially earlier signal of tubular or microcirculatory stress, especially 1–2 days prior to traditional AKI markers (e.g., sCr).
- FENa: This is widely known but often unhelpful in critically ill and diuretic-treated patients.
- FEU: This is recommended by KDIGO in diuretic settings, but recent studies show inconsistencies (particularly in older or septic patients).
- FEK: This is emerging as a reliable, sensitive tool for early AKI detection; it is especially valuable because it appears to be less influenced by standard ICU interventions (e.g., loop diuretics).

### 4.3. Risk Stratification Tools

Developing practical risk stratification tools and identifying early biomarkers for AKI in trauma ICU patients are crucial for timely diagnosis, personalized management, and improved outcomes.

The risk of AKI in this population is multifactorial, as previously described, and is impacted by trauma type, injury severity, hemodynamic instability (such as hypotension), and pre-existing comorbidities, including diabetes and cardiovascular disease. Specific injury patterns, such as thoracic and multiple traumas, are especially associated with AKI due to systemic inflammatory responses. Additionally, biomarkers like creatine kinase, sCK, which are indicative of rhabdomyolysis severity, are also linked with an increased risk of AKI. Many of these clinical factors and biomarkers are incorporated into various scoring systems designed to support early prediction and risk stratification of AKI in critically ill patients.

The Renal Angina Index (RAI) and modified RAI: The RAI was initially developed and validated in pediatric populations to facilitate early risk stratification for AKI within the first 72 h of admission to a pediatric ICU [338,339]. Inspired by the concept of angina pectoris in cardiology, “renal angina” highlights early indicators of renal injury. The modified Renal Angina Index (mRAI) adapts this tool for critically ill adults by incorporating clinical parameters such as diabetes, sepsis, mechanical ventilation, the use of vasopressors or inotropes, the percentage change in sCr from baseline, and fluid overload within the first 24 h of ICU admission. The mRAI, using a cutoff score ≥ 10, has shown superior predictive capability for AKI stage 2 or higher over the next 2–7 days when compared to sCr changes alone [340,341]. Another ICU-based study confirmed the mRAI’s utility in predicting moderate-to-severe AKI, revealing its applicability across various patient subgroups, including those with trauma. Furthermore, an mRAI score above 10 correlates with higher rates of in-hospital major adverse kidney events (MAKEs) [342].

McMahon score: Recognizing the importance of early risk stratification in rhabdomyolysis, the McMahon score was introduced in 2013 (Table 3). It utilizes admission parameters—such as serum creatinine kinase (sCK), initial creatinine, calcium, phosphate, and bicarbonate—to predict the need for RRT or the likelihood of in-hospital mortality. Scores below 5 carry a 3% risk of requiring RRT or experiencing death, whereas scores above 10 indicate a 52% risk [343]. A ten-year retrospective study found that a McMahon score of ≥6 is more sensitive, specific, and timely than monitoring peak sCK or its trajectory alone [107]. A clinical consensus statement from the Critical Care Committee in Trauma Surgery also recommends a McMahon score ≥ 6 to identify patients requiring high-volume fluid resuscitation or RRT, and these patients face elevated mortality risk [123]. Interestingly, although males are at higher risk for perioperative rhabdomyolysis, the original McMahon study suggested that females might experience worse outcomes, potentially because males often exhibit a milder presentation of the condition with elevated CK but without severe complications. Consistent with other findings, patients who developed AKI had significantly higher rates of head, neck, thoracic, and abdominal injuries, while extremity injuries were relatively less common [18,109].

Haines prediction model: In 2018, Ryan W. Haines and colleagues proposed a novel prediction model specifically designed to assess AKI risk in trauma ICU patients [344]. This model uses routinely available clinical variables, including age, SCr, serum phosphate, and the total number of packed red blood cell units transfused within the first 24 h. Its strong AUC-ROC underscores its predictive strength and bedside relevance for guiding early interventions and optimizing outcomes in trauma settings.

While the RAI and McMahon score target broader or more heterogeneous populations, the Haines model offers a more trauma-specific focus, filling a vital niche for AKI prediction in this subgroup. However, its utility depends on seamless integration into clinical workflows and confirmation across diverse trauma populations. Whereas the RAI is valuable for continuous risk monitoring, the McMahon score accounts for a broader array of rhabdomyolysis-related factors. In contrast, the Haines model provides a simpler, trauma-specific tool that complements rather than replaces existing risk stratification methods. These tools help personalize patient management, optimize fluid resuscitation, and enhance outcomes by enabling early detection and prevention of kidney injury in this vulnerable population. Table 3 shows a side-by-side comparison of AKI risk stratification tools—their required inputs, optimal timing windows, predicted endpoints (AKI onset, persistence, RRT, mortality), and key strengths and limitations.

### 4.4. Guidelines, Recommendations, and Clinical Integration of AKI Biomarkers in Critical Care

Current international guidelines emphasize that biomarkers should be integrated with, rather than replace, functional criteria in the diagnosis and management of AKI. KDIGO emphasizes that the kidney is resilient, so acute changes in sCr or urine output usually indicate significant systemic derangement and portend poor prognosis [345]. The guideline recommends stratifying patients for AKI risk based on susceptibility and exposure factors (e.g., sepsis, trauma, cardiac surgery, nephrotoxins, dehydration, CKD, diabetes) and then tailoring management accordingly. For those at increased risk, regular measurement of sCr and urine output remains the primary monitoring strategy, with frequency individualized to risk and clinical course. KDIGO acknowledges that biomarkers of kidney damage or stress (such as NGAL, KIM-1, TIMP-2·IGFBP7, DKK3, CCL14) may complement functional markers by identifying subclinical AKI, refining differential diagnosis, and predicting persistence or recovery. However, it stops short of recommending the universal use of biomarkers, instead encouraging their integration with clinical assessment in high-risk settings, especially perioperative care, sepsis, and nephrotoxin exposure [345]. The ADQI consensus (2020) [346] went further, issuing structured recommendations across risk assessment, prevention, diagnosis, management, and recovery, advocating a Kidney Health Assessment before high-risk exposures, integrating comorbidity profiles with biomarker testing, and proposing subdivisions of KDIGO stage 1 (1S, 1A, 1B) to capture biomarker-positive AKI earlier. The ADQI also highlighted specific markers for prognosis: TIMP-2 × IGFBP7 for imminent risk, CCL14 and DKK3 for persistent AKI and long-term dysfunction, and proenkephalin A as a filtration marker with predictive value in sepsis [347].

Critical care societies, including the European Society of Intensive Care Medicine (ESICM) and Intensive Care Medicine (ICM) (ESICM/ICM), endorse integration of biomarkers into ICU practice for early recognition, risk stratification, and differentiation of functional versus structural AKI, and trauma guidelines increasingly reinforce their use in high-risk patients exposed to ischemia–reperfusion, rhabdomyolysis, or nephrotoxins. Practical guidance now recommends that ICU physicians use biomarkers to triage high-risk patients after surgery or septic shock, clarify the meaning of creatinine “bumps” during decongestion or cirrhosis (e.g., uNGAL < 220 µg/g predicts terlipressin response), anticipate persistent AKI (CCL14, DKK3), and trigger KDIGO-based bundles focused on hemodynamic optimization, nephrotoxin avoidance, and drug stewardship [348].

Evidence from randomized controlled trials supports this targeted approach. The PrevAKI [349] single-center and multi-center trials showed that biomarker-guided KDIGO bundles decreased both overall AKI and stage II/III AKI after cardiac surgery. The BigPAK trial [350] in major noncardiac surgery revealed a reduction in severe AKI and shorter ICU and hospital stays in biomarker-positive patients randomized to strengthened prevention. Ongoing multi-center studies (BigPAK-II, PrevProgAKI) aim to confirm benefits across broader ICU populations. While mortality and dialysis outcomes remain less invariably improved, these trials provide proof of concept for biomarker-guided prevention [351,352].

A systematic review and health economic evaluation conducted by NICE (Brazzelli et al., 2022 [353]) evaluated the use of biomarker assays, such as NephroCheck (TIMP-2·IGFBP7) and NGAL platforms (Abbott ARCHITECT, BioPorto), in patients evaluated for admission to critical care. While meta-analyses have shown that these markers have some predictive value for AKI, the clinical evidence base is heterogeneous. It lacks trials directly linking biomarker-guided strategies to enhanced outcomes. The accompanying cost–effectiveness modeling demonstrated high uncertainty, with incremental quality-adjusted life-year (QALY) gains being very small and often negative and incremental costs varying across scenarios. Some analyses suggested potential cost savings with improved outcomes, but others indicated higher costs with reduced effectiveness, leaving no robust base-case incremental cost–effectiveness ratio. Overall, the report concluded that the current evidence is insufficient to support routine adoption of these tests in critical care and recommended restricting their use to research until stronger data demonstrate clinical benefit and economic value [353].

By contrast, more recent economic models indicate that biomarker-directed strategies, particularly those targeting CCL14 for persistent AKI, can be cost-effective by facilitating resource allocation, enabling earlier intervention, and preventing unnecessary dialysis [354].

Taken together, current guidance from KDIGO, the ADQI, the ESICM/ICM, and trauma societies supports the selective use of validated biomarkers in high-risk ICU patients to enrich risk assessment, detect subclinical AKI, predict persistence, and individualize preventive interventions. At the same time, economic evaluations underscore the importance of context-specific cost analyses. For ICU physicians, this means biomarkers are reasonably applied as adjuncts for early triage, therapy guidance, and prognosis, not as standalone diagnostics, with practice shaped by local availability, infrastructure, and health-system cost thresholds. At admission, risk scores such as the (RAI) or McMahon score provide a baseline framework for identifying patients at high risk of AKI. In those with significant exposures or early clinical suspicion, measurement of TIMP-2 × IGFBP7 enables the timely recognition of tubular stress and can trigger the early initiation of KDIGO preventive bundles. It is validated for identifying critically ill patients at imminent risk of moderate/severe AKI within 12–24 h. A cutoff ≥ 0.3 indicates increased risk warranting closer monitoring and preventive measures (KDIGO bundle), while ≥2.0 identifies very high risk. To refine etiology, biomarkers such as NGAL, KIM-1, and IL-9 help distinguish structural tubular injury or interstitial nephritis from purely functional creatinine rises, thereby preventing inappropriate management changes. For prognostication, CCL14, DKK3, and proenkephalin help anticipate the development of persistent AKI, guide the timing of renal replacement therapy, and identify patients who need structured post-ICU nephrology follow-up. Significantly, biomarker data should be interpreted within a multidisciplinary clinical context, incorporating hemodynamic status, imaging findings, and comorbid conditions, to confirm that they inform individualized and actionable care decisions rather than functioning as isolated tests.

## 5. Conclusions

In trauma ICU patients, serum creatinine and cystatin C remain late and often unreliable indicators of kidney injury, leaving clinicians without timely guidance during the critical early phase. The integration of newer biomarkers—such as NGAL, KIM-1, L-FABP, and TIMP-2×IGFBP7—offers earlier recognition of tubular stress and injury, while prognostic markers like CCL14 and DKK3 help identify patients at risk for persistent dysfunction and adverse outcomes. Functional electrolytes (UNa, FEK) and trauma-specific risk scores (mRAI, McMahon, Haines model) further enrich early risk stratification when interpreted in conjunction with the clinical context.

Looking ahead, the future of AKI care in trauma is likely to combine biomarker panels with validated risk tools and machine learning models, facilitating real-time prediction that adapts to patient heterogeneity. This integrated approach has the potential to move AKI management from delayed recognition to proactive prevention, permitting earlier nephroprotective strategies, more careful fluid and drug use, and structured follow-up of high-risk survivors. For clinicians, this signals a shift toward predicting AKI rather than reacting to it, with biomarkers and predictive analytics serving as practical decision support tools embedded into trauma critical care. Future work should focus on trauma-specific validation, harmonized thresholds, and pragmatic implementation pathways to integrate these tools into routine critical care practice.

## Figures and Tables

**Table 1:** Key biomarkers for early detection, risk stratification, and prognosis of trauma-related acute kidney injury.

Biomarker	Functional Group	Pathway/What it Reflects	Specimen	Key Caveats/Practical Notes	References
Creatinine	Filtration	↓ GFR (rises after ≥50% nephron loss)	Serum	Delayed 24–72 h; confounded by muscle mass and fluid shifts	[270,271,272,273,274]
Cystatin C	Filtration plus	Freely filtered low-MW protein; more muscle-independent	Serum	Influenced by steroids, thyroid status, ethnicity; rises earlier than creatinine	[275,276,277,278,279,280,281]
TIMP-2 × IGFBP-7 (NephroCheck™)	Early stress/cell cycle arrest	G1 cell cycle arrest in stressed tubular cells	Urine (20-min cartridge)	Repeat q12 h for 48 h; AUC ≈ 0.79 in trauma cohorts	[16,314,315,316]
NGAL	Early stress/injury	Tubular and neutrophil release within 2–3 h	Plasma and urine	Systemic inflammation or UTI can elevate baseline	[282,283,284,285,286,287]
L-FABP	Early stress/hypoxia	Tubular hypoxia and lipid peroxidation (appears ~2–4 h)	Urine	Also ↑ in liver and mesenteric injury; bedside semi-quant strip available	[301,303,304,307,308]
KIM-1	Structural injury	Shed from injured proximal tubule brush border	Urine	Baseline CKD and heavy-metal exposure reduce specificity	[269,292]
Interleukin-18 (IL-18)	Structural injury and inflammation	Inflammasome cytokine from proximal tubules	Urine	↑ in sepsis and TBI irrespective of AKI	[293,294,295]
Monocyte chemoattractant protein-1 (MCP-1/CCL2)	Structural injury and inflammation	Chemokine recruiting monocytes to injured tubules	Urine	Elevated in systemic inflammation; complements IL-18 in sepsis/trauma panels	[293,294,295]
CCL14	Persistence/prognosis	Chemokine signaling sustained tubular injury	Urine	Strong predictor of persistent stage 2–3 AKI	[296,297,298]
Dickkopf-3 (DKK3)	Stress/fibrosis risk	Wnt/β-catenin stress protein driving fibrosis	Urine/serum	High baseline marks susceptibility to AKI and future CKD	[299,300]
suPAR	Prognostic/systemic	Chronic immune activation and endothelial dysfunction	Plasma (EDTA) or serum	Upper quartile ≈ 3-fold AKI risk; not kidney-specific	[309,310]
CHI3L1/YKL-40	Prognostic/systemic	Macrophage-derived glycoprotein (acute phase)	Plasma	Additive value with suPAR in sepsis/trauma	[311,312]
Soluble TNF-R1/TNF-R2	Prognostic/monitoring	Systemic inflammatory load and endothelial activation	Plasma	Lab-developed ELISA only; good for serial trending	[318]
Mitochondrial DNA (mtDNA)	Cell-free DNA injury	DAMP released after cellular necrosis	Plasma	High admission levels predict AKI in trauma	[317]
Nuclear DNA (nDNA)	Cell-free DNA injury	Chromatin fragments from necrotic cells	Plasma	Early rise precedes creatinine; correlates with severity	[317]
Nucleosomes	Cell-free DNA injury	Histone–DNA complexes; severe tissue damage marker	Plasma	Mirrors nDNA; potential therapeutic target flag	[317]
CKD273 (peptidomic panel)	Proteomic classifier	273 urinary peptides (mostly collagen fragments)	Urine (CE-MS)	Early fibrosis/high-AKI-risk classifier	[319]
AKI204 (peptidomic panel)	Proteomic classifier	204 urinary peptides linked to tubular stress	Urine (CE-MS)	Predicts AKI and mortality in trauma cohorts	[319]

Filtration markers (creatinine) reflect glomerular filtration changes, whereas “filtration-plus” markers such as cystatin C rise earlier and are less muscle-dependent; “early stress/cell cycle arrest” proteins (TIMP-2 × IGFBP-7, NGAL, L-FABP) appear within hours of ischemia or nephrotoxin exposure, while “structural injury and inflammation” biomarkers (KIM-1, IL-18, MCP-1) originate from damaged tubular cells or inflammasomes. “Persistence/prognosis” markers (CCL14) forecast prolonged AKI; “stress/fibrosis risk” proteins (DKK3) signal kidneys primed for fibrotic remodeling; “prognostic/systemic and monitoring” markers (suPAR, YKL-40, soluble TNF-R 1/2, hs-CRP, fibrinogen) mirror systemic inflammation and are most useful when trended over time. “Cell-free DNA injury” markers (mtDNA, nDNA, nucleosomes) are damage-associated molecular patterns released after extensive cell death, and “proteomic classifiers” such as CKD273 and AKI204 are multipeptide urinary signatures generated by capillary electrophoresis–mass spectrometry (CE-MS). Abbreviations: q12 h = every 12 h; AUC = area under the ROC curve; EDTA plasma = plasma collected in ethylenediaminetetraacetic acid tubes; DAMP = damage-associated molecular pattern; hs-CRP = high-sensitivity C-reactive protein; and GFR = glomerular filtration rate. Approximate rise times after renal insult in adult trauma cohorts are 2–3 h for NGAL, 2–4 h for L-FABP, and ≤4 h for TIMP-2 × IGFBP-7, whereas creatinine lags 24–72 h and increases only after >50% functional reserve is lost. TIMP-2 × IGFBP-7 (NephroCheck™) is currently the only FDA-cleared test for AKI risk assessment; all other assays remain laboratory-developed or research-only.↓ decrease; ↑ increase.

**Table 3:** Tools for early AKI risk stratification in critically ill/trauma patients.

Score System	Target Population	Key Variables	Scoring/Cutoff	Primary Outcome(s) Predicted	References
Renal Angina Index (RAI)/Modified RAI (mRAI)	Pediatric (RAI) and adult critically ill (mRAI)	–RAI: change in serum creatinine, fluid overload, and clinical risk factors. –mRAI: diabetes, sepsis, mechanical ventilation, vasopressors/inotropes, % change in creatinine, and fluid overload.	mRAI cutoff ≥ 10.	Moderate-to-severe AKI (stage 2 or higher). Major adverse kidney events (MAKEs).	[338,339,340,341,342]
McMahon Score	Rhabdomyolysis (broadly including trauma)	Admission creatine kinase, initial creatinine, calcium, phosphate, bicarbonate, patient age, sex, and clinical origin (trauma vs. other causes).	<5: 3% RRT/death risk. ≥10: 52% risk.	Need for RRT and in-hospital mortality. High-volume fluid resuscitation needs.	[107,123,343]
Haines Prediction Model	Trauma ICU patients	Age, serum creatinine, serum phosphate, and number of PRBC units transfused within 24 h.	Continuous risk model; high AUC-ROC.	AKI risk in the early phase of trauma ICU admission.	[344]

## Data Availability

No new data were created or analyzed in this study. Data sharing is not applicable to this article.
